# Innovative Mucosal Vaccine Formulations Against Influenza A Virus Infections

**DOI:** 10.3389/fimmu.2019.01605

**Published:** 2019-07-17

**Authors:** Cynthia Calzas, Christophe Chevalier

**Affiliations:** VIM, UR892, Equipe Virus Influenza, INRA, University PARIS-SACLAY, Jouy-en-Josas, France

**Keywords:** influenza A virus, mucosal vaccines, adjuvant, delivery systems, intranasal immunization

## Abstract

Despite efforts made to develop efficient preventive strategies, infections with influenza A viruses (IAV) continue to cause serious clinical and economic problems. Current licensed human vaccines are mainly inactivated whole virus particles or split-virion administered via the parenteral route. These vaccines provide incomplete protection against IAV in high-risk groups and are poorly/not effective against the constant antigenic drift/shift occurring in circulating strains. Advances in mucosal vaccinology and in the understanding of the protective anti-influenza immune mechanisms suggest that intranasal immunization is a promising strategy to fight against IAV. To date, human mucosal anti-influenza vaccines consist of live attenuated strains administered intranasally, which elicit higher local humoral and cellular immune responses than conventional parenteral vaccines. However, because of inconsistent protective efficacy and safety concerns regarding the use of live viral strains, new vaccine candidates are urgently needed. To prime and induce potent and long-lived protective immune responses, mucosal vaccine formulations need to ensure the immunoavailability and the immunostimulating capacity of the vaccine antigen(s) at the mucosal surfaces, while being minimally reactogenic/toxic. The purpose of this review is to compile innovative delivery/adjuvant systems tested for intranasal administration of inactivated influenza vaccines, including micro/nanosized particulate carriers such as lipid-based particles, virus-like particles and polymers associated or not with immunopotentiatory molecules including microorganism-derived toxins, Toll-like receptor ligands and cytokines. The capacity of these vaccines to trigger specific mucosal and systemic humoral and cellular responses against IAV and their (cross)-protective potential are considered.

## Introduction

Despite progress in antiviral therapies, influenza viruses remain an important cause of respiratory tract (RT) infections in humans and animals worldwide (1). Influenza viruses are members of the *Orthomyxoviridae* family and are classified into four genera (A, B, C, D). Influenza A viruses (IAV), whose natural reservoirs are aquatic birds, can infect a broad spectrum of animal species including humans and poultry. Based on the molecular structure and genetic characteristics of the surface glycoproteins hemagglutinin (HA) and neuraminidase (NA), IAV can be categorized into 18 HA subtypes and 11 NA subtypes.

IAV have a negative-sense, single-stranded RNA genome consisting of 8 segments encoding for at least 17 viral proteins (1). Each segment is associated with the viral nucleoprotein (NP) and the three polymerase components, namely the polymerase basic protein 1 (PB1) and 2 (PB2) and the polymerase acidic protein (PA). These ribonucleoprotein complexes are encapsidated by the matrix protein 1 (M1) beneath an envelope composed of a lipid bilayer derived from the host plasma membrane where are embedded the surface glycoproteins HA, NA and the matrix protein 2 (M2). HA is responsible for the binding of the virus to sialic acid moieties at the host cell surface. HA is a trimeric glycoprotein and each monomer is composed of two domains, a globular head (HA1) and a stalk domain (HA2). HA1, exposed at the surface of the virion is subject to a high degree of antigenic variations. HA2, more conserved across IAV, is involved in various steps of the virus life cycle, including the fusion between the viral envelope and the endosomal host membrane. NA is a tetrameric glycoprotein which enzymatically removes sialic acid residues from the surface of infected cells, allowing the release of budding virions. M2 is a tetrameric protein acting as a proton-selective ion channel which triggers the uncoating of the viral ribonucleoprotein complexes necessary for the release of the viral genetic material into the host cytosol. Unlike HA and NA, the ectodomain of M2 (M2e) is sparsely expressed at the surface of the virion, less subjected to the host immune pressure and consequently more conserved across IAV (1).

Circulating IAV are continuously evolving, leading to the emergence of new strains expressing surface glycoproteins that have distinct antigenic properties (1). In particular, point mutations in the viral genome RNA result in the emergence of new strains responsible for seasonal epidemics (“antigenic drift”), and the co-infection of a host with multiple IAV strains can result in genetic reassortments responsible for the emergence of novel subtypes (“antigenic shift”) that can give rise to strains with pandemic potential. The disease severity caused by IAV infections depends on several parameters such as viral and host factors. In humans, the virus initially targets the mucosa of the upper RT (URT) (nose, pharynx), leading to dry cough, nasal discharge, rhinitis, pharyngitis and fever, and can eventually reach the lower RT (LRT) (trachea, bronchi, bronchioles, alveoli) resulting in fatal pneumonia in severe cases. Seasonal influenza infections, which are mainly caused by H1N1 or H3N2 IAV strains, are responsible for 3–5 million human cases of severe infections and 290,000–650,000 fatal cases annually, most often in young children, the elderly and immunocompromised individuals (2). Pandemic IAV infections affect a broader category of populations and cause atypical and more severe clinical symptoms (1). In aquatic birds, low pathogenic avian influenza viruses (LPAIV) typically cause asymptomatic infections. In poultry, infections with LPAIV can be asymptomatic or provoke low to mild pathophysiological damages to the respiratory, digestive and reproductive systems (resulting in a drop in egg production), while infections with highly pathogenic avian influenza viruses (HPAIV) are characterized by high morbidity and mortality rates. Outbreaks in domestic poultry result in massive culling to control the viral spread and are thus responsible for important economic losses (3).

## The Mucosal Immune Responses Against IAV Infections

The host RT is not only the initial point of entry and replication of IAV, but also the site of the host immune defenses. Components of the antiviral immune responses are located in the RT lining fluids and in the mucosa-associated lymphoid tissues (MALT) (4). Broadly, in mammals, the MALT of the RT include the nasopharynx-associated lymphoid tissues (NALT) located at the entrance to the nasopharyngeal duct in the URT (Waldeyer's ring in humans), the bronchus-associated lymphoid tissues (BALT) randomly distributed along the LRT but most consistently located at sites of bronchial tree bifurcation, and the draining lymph nodes (DLN). The NALT and BALT are globally composed of organized inductive sites, comprising B cell follicles and interfollicular T cell areas, as well as effector sites, consisting of the diffuse tissue of the lamina propria. The MALT of the RT are overlaid by a surface epithelium made of ciliated cells and various transporter cells located in the follicle-associated epithelium (FAE), such as microfold (M) cells, which transfer the luminal antigens (Ags) to the subepithelial cells (5). MALT are also detected in the avian RT (6). Over the last decade, considerable progress have been done in the understanding of the mechanisms of host mucosal immune responses against IAV (mostly in experimental mouse models of IAV infections).

At the first steps of infection, IAV must face host innate immune molecules with antiviral and/or immunomodulatory activities such as mucins, lectins, complement molecules, natural immunoglobulins (Ig) and antimicrobial peptides (7). Once the virus reaches and infects the epithelium, the local immune cells detect the viral components [also called pathogen-associated molecular patterns (PAMPs)] via pattern recognition receptors (PRRs) including Toll-like receptors (TLRs) (7). These cells produce various cytokines and chemokines involved in the antiviral defense and in the recruitment and activation of innate effector cells, which establish an antiviral program and prime adaptive immune responses.

Neutrophils, natural killer (NK) cells, and monocytes/macrophages interfere with viral replication and spread within the RT, and Ag-presenting cells (APCs) including dendritic cells (DCs) initiate specific adaptive immune responses (7–9). Murine DC subsets can be classified into CD11c^hi^ conventional DCs (cDCs), CD11c^lo^B220^+^ plasmacytoid DCs (pDCs) and inflammation-induced CD11b^+^Ly6C^+^ monocyte-derived DCs (moDCs). In mice, after capturing IAV Ags in the MALT of the RT, respiratory DCs (including CD103^+^CD11b^lo^ and CD103^−^CD11b^hi^ cDC subsets) acquire a mature phenotype, transport viral Ags to the DLN and simultaneously process the Ags and present Ag-derived peptides on class I or II major histocompatibility complex (MHC) molecules to naive CD8^+^ and CD4^+^ T cells, respectively (10–14). Beside migratory cDCs, CD8α^+^CD11b^−^ resident cDCs are also involved in the cross-presentation of IAV Ags to naive CD8^+^ T cells in the DLN (10, 15). Following IAV infection in mice, moDCs accumulate in the RT and they promote immune-induced pathology. However, their complete elimination is detrimental because they facilitate the optimal expansion of effector CD8^+^ T cells in the infected lung (16). Finally, pDCs are a potent source of type I interferons (IFNs), they are involved in the generation of virus-specific antibody (Ab)-secreting cells (ASCs) and they ensure a full-magnitude CD8^+^ T cell response (17–19). Subsets of CD11c^+^CD103^lo^ myeloid DCs (mDCs), including CD1c^+^ mDC1 and CD141^+^ mDC2 (equivalent to CD103^−^CD11b^hi^ and CD103^+^CD11b^lo^ mouse cDC subsets, respectively), and pDCs have been identified in the human RT. Studies have suggested that mDCs and pDCs could traffic to the site of IAV infection, but the functionality of the human DC subsets during IAV infection remains largely unknown (20).

Following primary IAV infection in mice, activated CD4^+^ and CD8^+^ T cells are detected in the DLN and spleen and the most highly activated and divided cell populations migrate to the airways and notably to the lamina propria of the MALT (21, 22). Activated IAV-specific CD8^+^ T cells differentiate into effector cytotoxic T lymphocytes (CTLs) which kill virus-infected cells (23, 24). Activated IAV-specific CD4^+^ T cells differentiate into various subsets which present heterogeneous antiviral functionalities and organ location, including T helper 1 (Th1), Th17 cells, follicular helper CD4^+^ T cells and CD4^+^ CTLs (25–27). Studies in humans have mainly examined IAV-specific T cell responses in the peripheral blood. Secondary responses are generally observed because most adults have encountered IAV Ags multiple times during infections and/or vaccinations. In the absence of specific Ab responses to newly-emerged IAV, the presence of pre-existing circulating cross-reactive CD4^+^ or CD8^+^ T cells exhibiting cytotoxic activities correlates with protection against experimentally or naturally mild H1N1 or H3N2 infections in humans (28–30). A recent study showed that rapid and robust IAV-specific CD8^+^ T cell recall responses correlated with early recovery of patients from severe H7N9 disease (30). Most T cell epitopes are highly conserved across IAV and are located on internal proteins such as NP, M1 or polymerase subunits, but they are also present in surface glycoproteins HA and NA, and in M2 (9, 28, 31–33).

Follicular helper CD4^+^ T cells support the generation and maintenance of the germinal centers (GCs) in the B cell follicles in cooperation with follicular DCs (34). In GCs, activated B cells, which have received helper signals from cognate CD4^+^ T cells, experience intense proliferation and undergo processes of class switch recombination, somatic hypermutation and affinity selection. GC reactions usually result in the generation of specific long-lived ASCs producing high affinity switched protective Abs and memory B cells. Activated B cells can also participate in early GC-independent reactions, mainly characterized by the rapid generation of low-affinity/specificity ASCs/memory B cells (34). Primary IAV infection in mice results in the development of specific ASCs which present site-specific kinetics and isotype distribution (35, 36). Anti-IAV Abs display a variety of functions depending on their isotype, specificity, affinity, concentration and post-translational modifications. Secretory IgA, which are polymeric IgA produced by IgA ASCs in the lamina propria and secreted to the mucosal surface, prevent infection of the epithelial cells via extracellular or intracellular immune exclusion. In addition, polymeric IgA can cross-react with IAV heterovariants (different IAV viruses belonging to the same subtype) or heterosubtypic strains (different IAV viruses belonging to different subtypes) (37). This breadth of reactivity could be linked to the avidity conferred by the polymeric form and/or the effect of the constant heavy chain in modulating the specificity/affinity of the variable regions (37, 38). IgA are thus an essential protective front line of defense against highly variable IAV. Whereas, IgG likely play a minor role in supporting secretory IgA in the prevention of IAV infection in the URT, they are crucially involved in the protection of the LRT in mice (39). In humans with low serum hemagglutination-inhibition (HAI) Ab titers, nasal and serum IgA provide protection against an i.n. experimental H1N1 challenge (40). However, the relative role of mucosal IgA vs. IgG in the (cross)-protection against IAV infection in humans remains poorly characterized.

IAV-specific Abs generated after an infection are mostly directed against the variable HA1 and, in a lesser extent, against NA (41). Anti-HA Abs mainly interfere with the virus infectivity by preventing virus binding to the sialic acid molecules and entry in epithelial cells. High HAI Ab titers are traditionally considered as the primary correlate of protection against IAV in humans and in animal models (1). Anti-NA Abs prevent the release of newly formed virions. Broadly neutralizing Abs directed against the conserved HA2 can occasionally be elicited in IAV-infected individuals and they interfere with the virus infectivity through several mechanisms (42). Besides neutralizing functions, IAV-specific Abs also exhibit Fc-related effector functions including Ab-dependent cellular cytotoxicity, Ab-dependent phagocytosis and Ab-dependent complement mediated-lysis (43–45). Abs directed against HA2, NA, M2e, NP, and M1 isolated from humans or animals have the potential to mediate protection via Fc-dependent mechanisms (43–45).

The clearance of a primary viral infection results in the establishment of long-lasting memory immune cells detectable in the MALT and in other lymphoid tissues, in the circulation and in the bone marrow. These cells play a critical role in the fight against reinfections. Among influenza-specific memory T cell populations, lung-resident memory CD8^+^ T (CD8^+^ T_RM_) cells are crucially involved in the heterosubtypic cross-protection against pulmonary infection in mice (46, 47). Virus-specific CD8^+^ T_RM_ cells can also be generated in the URT after IAV infection in mice. This cell population can efficiently clear a secondary heterosubtypic IAV infection from the URT and consequently blocks the spread of the virus from the URT to the LRT and the subsequent development of severe pulmonary diseases (48). IAV infection also stimulates the generation of specific lung CD4^+^ T_RM_ cells in mice (49) and adoptive transfer experiments demonstrate that these cells have a better protective potential against homologous reinfection than memory CD4^+^ T cells isolated from non-MALT locations (50). IAV-specific lung CD8^+^ T_RM_ cells are also detected in humans but due to practical and ethical limitations, the implication of mucosal T_RM_ cells in the resistance against IAV reinfection in humans is largely unknown (49, 51).

Whereas, long-lived ASCs serve as an immediate first line of defense against IAV homologous reinfections by secreting Abs with high specificity/affinity, memory B cells are more specialized to respond to antigenically divergent viruses. In particular, recent studies in mice have shown that GCs in the BALT select cross-reactive B cell repertoire, resulting in the generation of tissue-resident broad reactive memory B cells (52). The presence of these memory B cell populations at the site of infection facilitates their direct contact with intact influenza virions and could promote a faster production of high-affinity (cross-reactive) Abs through T-independent and PRR-dependent pathways (34). Ab responses induced by natural IAV infections in humans are relatively broad and long-lived (41). However, further investigations are needed to localize and identify cell subsets involved in these memory cross-reactive humoral responses (41).

## Problematic Issues Related to Current IAV Vaccines

Vaccination remains the most efficient and cost-effective means to protect human and animal populations against IAV (1). Most influenza vaccines available on the market are inactivated influenza A virus (IIV) vaccines administered via the parenteral route. Three types of IIV vaccines exist, namely whole inactivated virus (WIV) vaccines consisting of formaldehyde- or β-propiolactone-inactivated whole virion, split virus vaccines, and subunit vaccines. In split virus vaccines, the virus envelope is broken by diethyl ether or detergent treatment that disrupts the particulate organization and exposes all viral proteins, while subunit vaccines consist of surface proteins HA and NA separated from the nucleocapsid and lipids. While WIV preparations are commonly used in human pre-pandemic vaccines and in poultry, current human seasonal vaccines are mainly split virus or subunit vaccines. Current IIV vaccines predominantly induce virus-specific Ab responses directed against HA1 and do not stimulate efficient cellular immune responses. Thus, the efficiency of these vaccines is restricted to the protection against homologous/antigenically similar strains (1).

Beside IIV vaccines, live-attenuated influenza virus (LAIV) vaccines administered via the intranasal (i.n.) route are also available in humans. LAIV vaccines are composed of cold-adapted virus strain(s) restricted to the URT and causing only mild symptoms (1). LAIV and IIV vaccines are equally effective in adults, while studies have concluded that LAIV vaccines are more efficient in children, generating broader and longer-lived immune responses. Indeed, LAIV vaccines mimic the natural route of infection and consequently induce stronger mucosal IgA and broader T cell-mediated immune responses than IIV vaccines (1). A recent study in mice comparing two different licensed influenza vaccines showed that, in contrast to IIV vaccines injected parenterally, i.n. LAIV vaccines elicited the generation of lung T_RM_ cells, conferring long-term protection against various non-vaccine strains (53). Despite very promising results, some drawbacks have been associated with the use of LAIV vaccines (1). A suboptimal protection of LAIV vaccines in children against the 2009 H1N1 pandemic virus has been reported in the USA, which might be due to an impaired viral replication in the URT, resulting in a decreased stimulation of the host (mucosal) immune system. Also, for safety concerns, LAIV vaccines are contraindicated in children <2 years old and in immunocompromised individuals. Finally, the virus strain(s) composing LAIV vaccines could theoretically undergo genetic reassortments with circulating wild type viruses (1).

Thus, due to its ability to generate broadly reactive and long-term protective immune responses at the front line of virus entry, vaccination via the mucosal route is an appropriate strategy for the prevention and control of IAV infections. However, current mucosal vaccines need to be reformulated into safer, more refined and immunogenic preparations. In order to counteract the high variability of IAV, an active area of research area focusing on developing subunit vaccine candidates containing conserved surface or internal protein(s)/epitope(s) of the virus has garnered much interest. These “universal” vaccines aim at generating cross-reactive Abs and/or cross-reactive T cell responses (1). Alongside WIV, split or protein subunit vaccines, nucleic acid-based subunit vaccines induce vaccine Ag production in the host itself and can engage both humoral and cell-mediated immune responses (1, 31). Pre-clinical and clinical studies have shown that nucleic acid-based IAV vaccines can be efficiency delivered by live viral vectors through parenteral or mucosal routes and viral-vectored vaccines are commercially available for the control of avian IAV in poultry (54, 55). Nucleic-acid based vaccines delivered via non-lived vectors mimic infection or immunization with live microorganisms while being non-infectious, egg/cell-free manufacturable (ensuring a rapid, cost-effective and scalable production) and they do not have to address the challenge of preexisting or induced anti-vector immunity which could prevent repeated immunizations (56–58).

## Adjuvants/Delivery Systems for Intranasal Vaccination Against IAV Infections

The goal of vaccination is to generate potent and long-term protective immune responses against infections. Unlike attenuated live vaccines, inactivated vaccines (especially purified or recombinant subunit vaccines) usually require additional compounds to be effective. The role of an adjuvant is to increase vaccine efficacy by modeling the quality and the quantity of the host immune responses. In particular, the adjuvant can affect the magnitude, breadth, specificity, affinity, kinetics, longevity, and composition of the immune responses (59–61). The incorporation of adjuvant(s) into a vaccine formulation can decrease the amount of the vaccine Ag(s) and the number of doses required to induce protective immunity, and enhance vaccine efficacy in special populations (the elderly, neonates/infants, immunocompromised individuals) (59–61). Adjuvants were formerly classified as delivery systems, which function as vehicles to which the vaccine Ag(s) can be associated, and immune potentiators. However, there is now evidence that some delivery systems also exhibit immunostimulatory properties. Adjuvants exert their effect through various mechanisms including the formation of a depot at the site of injection, which guarantees a slow release of the vaccine Ag(s) and a constant stimulation of the host immunity (depot effect), the recruitment of immune cells, the activation of innate immune receptors, the enhancement of Ag uptake by APCs and the activation and the maturation of APCs (59–61). By targeting innate immune cell subsets and by generating a particular cytokine milieu (Th1, Th2, and/or Th17 for example), adjuvants can shape the nature of the subsequent adaptive immune responses to produce the most effective and protective reaction against a given pathogen.

**Figure 1:**
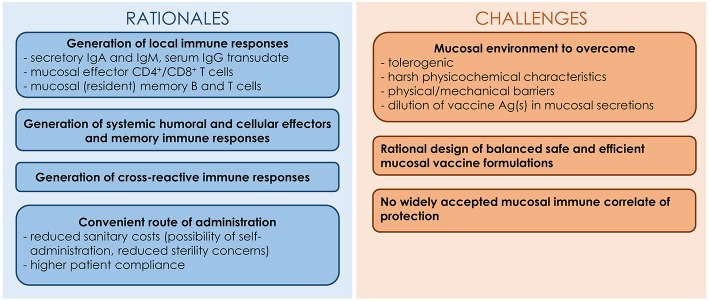
Rationales and challenges for the development of IAV mucosal vaccines.

Despite the relevance of the mucosal route of vaccination in the fight against respiratory pathogens, this method of administration faces several hurdles (62). In addition to encountering a tolerogenic mucosal environment, vaccine Ag(s) must resist degradation caused by the harsh mucosal environment characterized by the presence of proteases, nucleases and low pH. In addition, the Ag(s) is (are) likely to be diluted in the mucosal fluids and swept away by the mucociliary clearance. Consequently, mucosal vaccines have to be carefully formulated with adjuvants in order to breach the host mucosal barriers and allow the vaccine Ag(s) to reach and stimulate the cells of the underlying MALT (62). Mucosal adjuvants should ensure the integrity and the stability of vaccine Ag(s), exhibit mucoadhesive properties and allow Ag uptake by epithelial cells and/or M cells. The rationale and challenges for the development of IAV mucosal vaccines are presented in Figure 1.

In this section, we have compiled the delivery/adjuvant systems tested for i.n. administration of IIV vaccines with a special emphasis on experimental animal studies (see Supplemental Table 1). We have focused our work on non-replicative delivery/adjuvant systems (excluding viral and bacterial vectors).

### Bacterial Enterotoxins and Derivatives

The most potent and studied mucosal adjuvants in pre-clinical studies are cholera toxin (CT), the closely related *Escherichia coli* heat-labile toxin (LT) and their derivatives (detoxified/non-toxic holotoxins, isolated subunits). CT and LT holotoxins are composed of a pentameric B subunit, which binds to GM1-ganglioside receptors ubiquitously present on the surface of most nucleated cells, non-covalently associated with an A subunit. The A1 portion of the A subunit enzymatically ADP-ribosylates the α subunit of the GTP-binding protein Gs (Gsα), causing a dramatic elevation of intracellular cyclic AMP responsible for the efflux of ions and water from the targeted cells associated with watery diarrhea. The mucosal adjuvant properties of enterotoxins rely on a better accessibility of the co-administered Ag(s) to the cells of the MALT, which is related to an increased permeability of the mucosal barriers and/or to an enhancement of the recruitment and activation of local APCs (63).

Experimental studies in mice have demonstrated that these holotoxins are potent immunostimulators for mucosal IIV vaccines (64, 65). For example, the addition of CT to H1N1 WIV vaccine administered to mice via the i.n. route enhanced the magnitude of the serum and mucosal Ab responses directed against homologous, H1N1 heterovariant and H3N2 heterosubtypic viruses (65). CT increased the breadth of protection of the vaccine and all mice receiving the adjuvanted formulation survived the i.n. H3N2 heterosubtypic challenge (65). The adjuvant also stimulated the generation of long-lived ASCs lodged in the bone marrow that secreted IgA and IgG reactive against the homologous and the H3N2 heterosubtypic viruses (65). Holotoxins engineered into less toxic molecules, through site-directed mutations of the enzymatically active A subunit, and administered with influenza WIV or subunit vaccines also retained adjuvant functions (66, 67). However, the i.n. delivery of vaccines formulated with native or even detoxified LT in humans resulted in the development of facial paralysis (Bell's palsy) in some individuals, stopping further clinical use of enterotoxin-based adjuvants (63).

Enterotoxins lacking B subunit are good alternatives to i.n. holotoxin-based adjuvants because they keep strong adjuvant functions without toxic side effects (68, 69). For example, a study in mice evaluated the ability of the A1 subunit of the CT (CTA1) to potentiate the immunogenicity of a subunit vaccine composed of a portion of HA2 of a H5N1 virus [HA2_H5N1(15−137)_] fused with an M2 consensus sequence spanning the residues of the extracellular and cytoplasmic domains of M2 from H1N1, H5N1 and H9N2 viruses (consM2_H1N1/H5N1/H9N2_) (69). The fusion of CTA1 with the consM2_H1N1/H5N1/H9N2_-HA2_H5N1(15−137)_ chimeric protein increased the magnitude and persistence of M2/HA2-specific serum and mucosal humoral responses and M2/HA2-specific cellular immune responses in the spleen. Mice immunized with the CTA1-adjuvanted vaccine showed a better resistance to an i.n. challenge with divergent subtypes of IAV (H1N1, H5N1, H5N2, H7N3, H9N2) than mice immunized with the vaccine without CTA1 (69).

The chimeric protein composed of CTA1 fused with a synthetic dimer of the Ig binding D domain (DD) from *Staphylococcus aureus* protein A (CTA1-DD) is also a promising adjuvant for next generation mucosal vaccines against IAV. The DD portion of the molecule targets the B cell receptor (BCR) as well as the complement receptor CD21 on follicular DCs, resulting in an enhancement of GC formation (63, 70). In addition, experiments in mice and non-human primates have proven the safety of CTA1-DD (63). In pre-clinical tests, CTA1-DD increased the immunogenicity and/or the protective potential of various influenza subunit vaccines administered intranasally, either in fusion with M2e epitopes (71, 72), admixed with virus-like particles (VLPs) exposing M2e epitopes (73), or incorporated into lipid-based particles with IAV Ags (70). For example, the incorporation of CTA1-DD into immune stimulating complexes (ISCOMs) containing H1N1 HA and NA boosted the specific serum and mucosal humoral responses as well as type 1/type 2-cell-mediated immune responses in the spleen in mice. The immunostimulatory effects of CTA1-DD depended both on the enzymatic activity of the toxin and on B cell targeting (70). These encouraging results make CTA1-DD a very attractive adjuvant for mucosal anti-IAV vaccines for clinical or veterinary use (63).

### Flagellin

Flagellin, the primary structural component of the bacterial flagellum, is another promising mucosal adjuvant for anti-IAV vaccines. Flagellin targets TLR5 expressed at the surface of various cells including airway epithelial cells, DCs and lymphocytes, as well as cytosolic detectors of the NAIP (nucleotide oligomerization domain-like receptor (NLR) family, apoptosis inhibitory protein) family proteins (74). The mucosal adjuvant properties of flagellin rely on the acceleration of the transepithelial transport of the co-administered Ag(s) by the FAE and on the stimulation of the migration of subepithelial DCs into the FAE (75).

The i.n. co-administration of recombinant flagellin derived from *Vibrio vulnificus* or *Salmonella typhimurium* with H1N1 WIV vaccine or a trivalent split vaccine composed of two IAVs (H1N1 and H3N2) and an influenza B strain (IBV) boosted the immunogenicity of the vaccine in mice (76, 77). In particular, the adjuvant increased the serum and/or mucosal Ab responses and elicited systemic type 2-biased-cell-mediated immune responses (76, 77). The incorporation of flagellin into the vaccine preparation enhanced protection of mice against a subsequent i.n. homologous challenge (76, 77). The adjuvant exhibited a safer profile than enterotoxins because it did not accumulate in the central nervous system (77).

Flagellin is also a potent mucosal adjuvant for influenza subunit vaccines (78–80). The association of flagellin with IAV epitopes, as free fusion proteins or as membrane-anchored form into influenza VLPs, significantly increased the immunogenicity and the (cross)-protective potential of these vaccines against IAV in mice (78–80). For example, the membrane incorporation of flagellin into H1N1 (HA/M1) VLPs enhanced serum and mucosal Ab titers and elicited systemic type 1/type 2-cell-mediated immune responses specifically directed against the homologous H1N1 strain (78). Moreover, the addition of flagellin to the vaccine formulation induced higher cross-neutralizing Ab titers to a H3N2 virus detectable in the serum and in the lung lavage (78). Accordingly, mice immunized with the adjuvanted vaccine were all resistant to the i.n. H3N2 heterosubtypic challenge in contrast to the unadjuvanted group (78). Another *in vitro* study showed that the conjugation of flagellin on the surface of gold nanoparticles bearing a recombinant trimeric HA derived from a H3N2 virus stimulated the uptake of the vaccine by murine bone marrow-derived DCs and promoted the activation and the maturation of the cells (81). Mice intranasally immunized with such flagellin-adjuvanted vaccine developed higher influenza-specific immune responses and showed a better resistance against an i.n. homologous challenge (81, 82).

Interestingly, flagellin also significantly boosted the immunogenicity and the protective capacity of IIV vaccines in chickens, and notably when it was admixed with H5N2 WIV vaccine or fused with an epitope located in HA1 of a H7N9 virus (83, 84).

Clinical trials have proven the safety and the immunogenicity of flagellin-adjuvanted influenza subunit vaccines when administered via the parenteral route (85, 86). Further clinical investigations on the efficacy of this adjuvant in mucosal anti-IAV vaccination are needed.

### Proteosome-Based Adjuvants

Proteosomes and Protollin are two other potent mucosal immunoactivators. Proteosomes are nanoparticles composed of a mixture of outer membrane proteins and traces of lipooligosaccharides derived from *Neisseria meningitidis* and Protollin consist of proteosomes complexed with *Shigella flexneri* lipopolysaccharides. These adjuvants stimulate Ag uptake by the FAE and cells of the MALT, and promote the maturation and activation of APCs via the engagement of various PRRs (87). Pre-clinical tests in mice concluded that proteosomes and Protollin were safe and strong mucosal adjuvants for split (88, 89) or subunit (89, 90) IAV vaccines. In addition, recent clinical trials showed that an i.n. proteosome-adjuvanted trivalent split vaccine was safe, immunogenic, and effective in healthy adults (91). The vaccination protected subjects against illness following an experimental H3N2 homologous challenge, and the protection was correlated with pre-challenge specific serum HAI Ab and nasal IgA titers (91). Thus, this class of adjuvants deserves to be harnessed for the development of mucosal anti-IAV vaccination.

### Bacterial-Derived Vesicles

#### Bacterium-Like Particles

Bacterium-like particles, also known as Gram-positive enhancer matrix, are non-living microparticles derived from non-pathogenic food-grade bacteria *Lactococcus lactis*, and consist of bacterial-shaped peptidoglycan spheres deprived of intact surface proteins and intracellular content. The immunostimulating properties of bacterium-like particles are related to their ability to activate and stimulate the maturation of APCs (92). In mice, bacterium-like particles admixed with H1N1 or H3N2 split vaccine enhanced serum and mucosal humoral responses as well as systemic type 1-biased cellular responses directed against the vaccine strain (93, 94). Moreover, the presence of the particles in the vaccine formulation significantly increased the level of protection of mice against an i.n. homologous challenge (94). The adjuvanticity of bacterium-like particles was suggested to be TLR2-dependent (95). Successful pre-clinical tests were also reported using bacterium-like particles non-covalently coupled with purified influenza epitopes (HA, M2e or NP) (92).

Finally, a phase I clinical trial has proven the safety and the immunogenicity of i.n. vaccine formulations composed of seasonal influenza trivalent split vaccine mixed with bacterium-like particles (92). In particular, the adjuvant boosted the specific serum HAI Ab and nasal IgA titers and increased the frequency of IFN-γ-producing peripheral blood mononuclear cells (92).

#### Outer Membrane Vesicles

Outer membrane vesicles are nanosized vesicles naturally produced by Gram-negative bacteria (96). They are composed of various PAMPs such as lipopolysaccharides, lipoproteins, peptidoglycan, flagellin monomers and nucleic acids, and they can stimulate the activation of respiratory CD103^+^ DCs (96). The incorporation of outer membrane vesicles into a trivalent split vaccine boosted influenza-specific serum IgG and HAI Ab titers as well as IgG and IgA titers in the LRT in mice receiving the vaccine formulation via the i.n. route (96). In addition, the adjuvant stimulated type 1-cell-mediated immune responses in the lungs and spleen (96). All mice survived an i.n. homologous challenge and were significantly more resistant to H1N1 heterovariant or H5N2 heterosubtypic challenges than mice immunized with the unadjuvanted vaccine (96).

### Lipid-Based Adjuvants

#### Liposomes

Liposomes are self-assembling bi- or multi-layered lipid vesicles with an aqueous core ranging from 10 nm to several μm in diameter (97). Various categories of lipids can be incorporated into liposomes such as phospholipids, sterols (cholesterol) or sphingolipids. Liposomes are common vaccine delivery vehicles because they protect the associated Ag(s) from the degradation or neutralization, they exhibit Ag depot effect and they are uptaken by APCs (97). Several physicochemical features of the liposomal formulations determine their immunomodulatory potentials including the lipid characteristics and the lipid composition of the liposomes (97). For example, the length and the degree of saturation of the hydrophobic tail of the lipids govern the fluidity/permeability and thus the stability of the particles. Cholesterol commonly modulates the stability of liposomes. In addition, the hydrophilic headgroups of the lipids determine the surface charge and, by extension, the ability of the liposomes to adhere/penetrate epithelial surfaces (in the context of a mucosal vaccination). The localization of the vaccine Ag(s) in the preparation, e.g., admixed with the liposomes, attached to the surface of the liposomes or encapsulated in the aqueous core of the liposomes, also influences the Ag-specific immune responses. Finally, the adjuvanticity of the liposomes can be improved by the incorporation of muco-adhesive/muco-penetrating polymers or PRR ligands into the formulation (97).

Both pre-clinical and clinical trials have demonstrated the remarkable adjuvant potential of liposomes in parenteral vaccination against IAV. Encouraging results have also been obtained in the context of mucosal vaccination (97, 98). Early studies showed that anionic dimyristoyl phosphatidylcholine (DMPC)/dimyristoyl phosphatidylglycerol (DMPG) liposomes facilitated the association and uptake of co-administered molecules by macrophages *in vitro* (99). The co-encapsulation of H1N1 split vaccine in DMPC/DMPG liposomes with synthetic oligodeoxynucleotides composed of unmethylated CpG motifs (CpG-ODN, TLR9 ligands) boosted the magnitude of virus-specific IgG and IgA titers in the URT and LRT and induced systemic type 1-biased cell-mediated immune responses in intranasally immunized mice (99). In addition, mice receiving the liposome-formulated vaccine showed reduced lung viral loads after an i.n. heterovariant challenge (99). CAF01, another liposomal preparation containing the monocationic lipid dimethyldioctadecylammonium bromide (DDAB) and the immunostimulator α,α'-trehalose 6,6'-dibehenate was effectively uptaken by murine bone marrow-derived DCs *in vitro* and stimulated the maturation of the cells (100). By using the human bronchial epithelial Calu-3 cell culture model, CAF01 was shown to enhance the transport of co-administered Ag through the mucus layer and across the epithelial cells (101). Accordingly, in mice, the i.n. administration of CAF01 with H3N2 (100) or seasonal trivalent (101) split vaccines remarkably strengthened virus-specific mucosal and systemic immune responses. Finally, polycationic liposomes composed of ceramide carbamoyl-spermine (CCS) sphingolipids complexed with cholesterol enhanced the efficacy of H1N1 monovalent or seasonal trivalent split vaccines (102, 103). Mice intranasally immunized with the vaccine adjuvanted with CCS/cholesterol liposomes showed an increase in serum and mucosal HAI Ab titers directed against homologous or heterovariant viruses (102, 103). In addition the adjuvant increased type 1-cell-mediated immunity in the spleen (102, 103). Finally, the animals exhibited a better resistance to i.n. homologous or heterovariant challenges than mice immunized with the unadjuvanted vaccine (103). CCS/cholesterol liposomes presented similar adjuvant effects to CT, while having a better safety profile (103). Interestingly, CCS/cholesterol liposomes had stronger adjuvant properties than anionic DMPC/DMPG or monocationic dioleoyl-3- trimethylammoniumpropane (DOTAP)/cholesterol liposomes, which could be linked to an extended retention time of influenza Ags in the nasal cavity and in the lungs (102, 103). Finally, CCS/cholesterol liposomes also enhanced the immunogenicity of H1N1 split vaccine in aged mice, albeit with lower specific serum and mucosal Ab titers than those observed in adult mice (103). Clinical trials with i.n. liposome-based influenza split vaccine have been reported (97, 104).

Some studies have demonstrated that liposomes are also potent mucosal carriers for influenza subunit vaccines in mice (105–107). For example, an M2e consensus sequence incorporated into the lipid bilayer of liposomes composed of a mixture of phospholipids, cholesterol and monophosphoryl lipid A (MPLA) generated M2e-specific serum IgG and systemic type 1/type 2-cell mediated immune responses in mice vaccinated subcutaneously and intranasally (107). The animals were also significantly protected against an i.n. homologous challenge (107). Interestingly, the M2e-specific serum Ab response inhibited *in vitro* viral cell lysis by various IAV subtypes including H2N2, H3N2, H6N2, H5N9, and H1N1 IAV, highlighting the potential of liposomes as mucosal delivery platforms for broadly-protective vaccines against IAV (107).

Preclinical and clinical studies have also concluded that cationic liposomes are attractive delivery platforms for nucleic-acid based IAV vaccines. Cationic lipids are known to efficiently complex the nucleic acids, facilitate cellular uptake and allow endosomal escape of nucleic acids into the host cytoplasm (57). The encapsulation of plasmid DNA encoding a HA protein derived from a H1N1 strain into cationic liposomes administered to mice via the i.n. route boosted the immunogenicity of the vaccine and all animals survived a subsequent i.n. homologous challenge unlike mice vaccinated with the naked plasmid DNA (105). Over the past decade, major technological advances have enabled mRNA vaccines to become promising candidates against IAV infections (57, 58, 108). In contrast to plasmid DNA, mRNA-based vaccines are delivered directly to the cytoplasmic site of function, eliminating the potential risk of integration into the host chromosome. The engineering of mRNA sequence and the development of separation and/or purification techniques can increase mRNA translation and stability and modulate its inherent immunogenicity (57, 58, 108). In addition, several delivery systems have been explored to improve the efficiency of mRNA vaccines, including liposomes (109). These carriers are usually composed of an ionizable cationic lipid, cholesterol, phospholipids, and lipid-linked polyethylene glycol which increases the half-life of the formulation (109). Liposome-encapsulated mRNA vaccines encoding IAV Ags such as HA, NP and/or M1 administered via intramuscular or intradermal routes generated significant B and/or T cell immune responses and conferred protection against i.n. homologous or heterosubtypic IAV challenge in various experimental animal models including mice, ferrets and/or non-human primates (110–112). A recent clinical study showed that mRNA vaccine encoding a HA protein derived from H10N8 or H7N9 viruses formulated with liposomes and administered via the intramuscular route was safe and immunogenic (111). While lipid nanoparticles are potent delivery systems for nasal mRNA vaccines in the context of anti-tumor vaccination (113), additional studies are needed to evaluate the efficacy of such carriers in mucosal vaccination against IAV.

#### Virosomes

Virosomes are a special category of liposomes which are composed of purified or synthetic lipids and viral envelope proteins such as HA and NA (“influenza virosomes”) (98). Influenza virosomes are commercially available, safe and efficient vaccine platforms for parenteral vaccination in humans. However, there have been no new i.n. formulations on the market since the withdrawal of LT-adjuvanted virosomal vaccines (98). Among various strategies to potentiate the immunogenicity of i.n. virosomal vaccines against IAV, the incorporation of cyclic di-nucleotides (CDN) (see section Nucleotide-Based Adjuvants) has proven to be valuable in pre-clinical tests (114). Mice immunized with H5N1 virosomes admixed with cyclic-di-adenosine monophosphate (c-di-AMP) developed stronger, broader and more persistent anti-IAV immune responses than mice immunized with the unadjuvanted virosomes (114). In particular, the animals showed higher mucosal IgA titers and higher serum HAI Ab titers reactive against homologous and heterovariant viruses. Moreover, the adjuvant increased the frequency of long-lived IgG ASCs lodged in the bone marrow. Mice also exhibited stronger systemic type 1/type 2/type 17 cellular immune responses, including higher frequency of multifunctional influenza-specific CD4^+^ T cells in the spleen (114). Finally c-di-AMP promoted protection of mice against i.n. homologous challenge (114). Clinical trials with virosome-based influenza vaccines administered via the nasal route are ongoing (98, 115).

#### Immune Stimulating Complexes (ISCOMs)/ Immune Stimulating Complex Matrix ISCOMATRIX

ISCOMs are negatively-charged pentagonal dodecahedrons with a ring-like stable structure about 40 nm in diameter which are spontaneously formed after mixing the vaccine Ag(s) with cholesterol, phospholipids and saponins from the *Quillaja saponaria* Molina tree (QuilA) which are potent immunostimulators (98). The corresponding structure without incorporated vaccine Ag is called ISCOMATRIX. ISCOM/ISCOMATRIX adjuvants are efficiently processed by various APCs and are thus widely-exploited vaccine delivery systems. The high affinity between saponins and cholesterol ensures the stability of the adjuvant (116). Vaccine formulations with more refined fractions of QuilA saponins have been developed in order to increase the safety and the tolerogenicity of the adjuvant (117). Pre-clinical studies have shown that this category of adjuvants markedly increases the immunogenicity and the protective potential of i.n. split or subunit IIV vaccines, with the viral Ags incorporated into ISCOMs or simply admixed with the ISCOMATRIX (70, 117, 118). Notably, mice intranasally immunized with an ISCOM/ISCOMATRIX-formulated H1N1 split vaccine presented higher nasal/lung IgA and serum IgG titers than mice vaccinated with the non-adjuvanted vaccine (118). Two other studies indicated that mice receiving envelope Ags derived from H1N1 virus in ISCOM/ISCOMATRIX formulations via the i.n. route exhibited stronger serum and mucosal Ab responses as well as higher systemic type 1/type 2 cellular immunity than mice receiving the free Ags (70, 117). The immunogenicity of ISCOM/ISCOMATRIX preparations can be boosted by the inclusion of immunostimulators (117). The addition of CTA1-DD into ISCOM/ISCOMATRIX formulations resulted in generating attractive and versatile mucosal delivery platforms for IAV Ags by targeting both DCs and B cells (117). Of note, a CTA1-3M2e-DD/ISCOM preparation could be kept 1 year at 4°C or as freeze-dried powder without altering the immunogenicity of the vaccine (117). ISCOMs are thus promising adjuvant for the development of cold chain-independent vaccines. Finally we can mention that ISCOMATRIX is also a suitable mucosal adjuvant for H5N1 WIV vaccines in chickens (119). Clinical trials with i.n. ISCOM-based influenza vaccines are currently under way (98, 116).

#### Other Lipid-Based Adjuvants

Surfacten is a modified bovine pulmonary surfactant forming unilamellar vesicles about 300–1,000 nm, which is widely used in premature babies with respiratory distress syndrome without significant adverse effects. It also displays potent mucosal adjuvant properties toward influenza split vaccines in mice (120, 121). More precisely, Surfacten enhanced the uptake of influenza Ags by bone marrow-derived DCs *in vitro*, and increased the distribution and the retention time of influenza Ags as well as the maturation of CD11c^+^ cells in the nasal cavity *in vivo* (120, 121). The adjuvant boosted both virus-specific local and systemic humoral and cell-mediated immunity and enhanced the protection of mice against i.n. homologous or heterovariant challenge (120, 121). Three major lipids, namely dipalmitoyl phosphatidylcholine (DPPC), phosphatidylglycerol (PG) and palmitate, and the surfactant protein C (SP-C) play an essential role in the adjuvant properties of Surfacten (121).

SF-10, a synthetic surfactant composed of DPPC/PG/palmitate lipids, a cationic SP-C-related peptide and a mucoadhesive carboxyvinyl polymer (CVP) is also a safe and effective delivery vehicle for IAV split vaccines in mice and non-human primates (122–124). *In vivo* experiments in mice showed that SF-10 stimulated the delivery of co-administered Ags to epithelial cells and APCs localized in the NALT, including CD8^+^CD11c^+^ DCs known to be involved in the cross-priming of CD8^+^ T cells (123). Accordingly, the i.n. administration of SF-10 with H1N1 split vaccine strengthened virus-specific serum and mucosal Ab responses and T cell-mediated responses (122, 123). In particular, mice immunized with the SF-10-supplemented vaccine showed higher frequency of IFN-γ- and IL-4-secreting lymphocytes in the NALT and CD8^+^ CTLs in the spleen (122, 123). These mice were also more resistant to an i.n. heterovariant challenge than those immunized with the unadjuvanted vaccine, and the protection was significantly reduced after depletion of CD8^+^ or CD4^+^ T cells (123). The resistance was associated with higher cytolysis activities in the lungs and DLN in the early phase of infection (123). Finally, young cynomolgus monkeys immunized with H1N1 split vaccine admixed with SF-10 via the i.n. route developed humoral immune responses composed of nasal Abs which cross-reacted against H1N1 heterovariant and H3N2 heterosubtypic viruses, and serum HAI Abs (124). Moreover, the animals exhibited influenza-specific immunological memory (124). SF-10 is thus a very promising adjuvant for i.n. influenza vaccines intended for humans, and in particular for young children.

Endocine is an anionic adjuvant based on mono-olein and oleic acid lipids found ubiquitously in the human body. Clinical vaccine studies against diphtheria and the human immunodeficiency virus (HIV) have proven that Endocine is a safe and well-tolerated i.n. adjuvant (125). Several studies have demonstrated that it is also a promising mucosal adjuvant for IAV vaccines (125–127). The addition of Endocine to an i.n. split trivalent vaccine enhanced both local and systemic cross-reactive humoral and cell-mediated immune responses in adult mice (126). The adjuvant is also a potent immunoenhancer in immunocompromised mice (127). Aged mice immunized with an Endocine-adjuvanted H1N1 split vaccine exhibited increases in influenza-specific serum IgG and HAI Ab titers and lung IgG and IgA titers (127). Finally, ferrets intranasally immunized with H1N1 split or WIV vaccine formulated with Endocine developed serum HAI and neutralizing Ab responses against homologous and H1N1 heterovariant viruses (125). Animals challenged intratracheally with the homologous strain were significantly protected from virus replication in the URT and LRT (125). Further (pre-)clinical investigations on the use of Endocine in i.n. IIV vaccine formulations are warranted.

Among other lipid-based adjuvants which have proven to be successful in mucosal vaccination against IAV in (pre)-clinical tests, we can cite the oil-in-water nanoemulsion W_80_5EC, consisting of a highly refined soybean oil combined with non-ionic and cationic surfactants and ethanol. *In vitro* and *in vivo* studies in mice suggested that the adjuvanticity of W_80_5EC was related to its ability to increase the uptake of the antigenic payload by nasal epithelial cells, facilitating the subsequent engulfment of Ag-primed apoptotic epithelial cells by DCs, followed by activation and migration of DCs to the DLN (128, 129). The adjuvant activity of W_80_5EC is related, at least in part, to TLR2/TLR4 activation (130, 131). Accordingly, W_80_5EC enhanced the immunogenicity and the protective potential of i.n. H1N1 WIV vaccine in mice (132). The i.n. administration of W_80_5EC with a seasonal IAV split vaccine also boosted serum humoral responses directed against homologous and heterovariant viruses in ferrets (133). Finally, a clinical study conducted with healthy human adults revealed that W_80_5EC was safe, well-tolerated and boosted the influenza-specific serum and mucosal Ab responses when combined intranasally with a seasonal trivalent split vaccine (134). A recent study mentioned that an mRNA vaccine expressing a HA protein derived from a H1N1 virus formulated with an oil-in-water cationic nanoemulsion elicited broad and protective immune responses against homologous and heterologous challenge when delivered intramuscularly to mice or ferrets (135). However, the efficiency of such formulations in the context of a mucosal immunization remains to be evaluated.

### Virus-Like Particles (VLPs)

VLPs are multimeric cage-like structures consisting of self-assembled structural viral proteins around a hollow interior space devoid of viral genetic material. VLPs mimic virions in shape, size, and molecular organization while being non-replicating and non-infectious (136, 137). VLPs can interact with APCs of the innate immunity. In addition, VLPs display highly repetitive epitopes and can stimulate B cell responses by BCR cross-linking (136).

Several pre-clinical studies have demonstrated that VLPs are potent i.n. delivery vehicles for IAV epitopes (79, 138). Influenza VLP vaccines are mostly produced by the recombinant baculovirus/insect cell expression system and consist of self-assembled M1 molecules surrounded by the lipid membrane derived from the insect cells where various combinations of influenza epitopes are anchored, including HA, NA and/or M2(e) (136, 137). Most of influenza VLPs fully protect animals against an i.n. homologous challenge, and several studies have reported their efficacy against heterologous viruses as well (136, 137). For example, the i.n. immunization of mice with VLPs incorporating H1N1 HA, NA and M1 [(HA/NA/M1) VLPs] induced the generation of IgG and IgA in the serum, the URT and LRT, which cross-reacted with a H5N1 virus (139). Mice were fully protected against a subsequent i.n. homologous challenge and exhibited a partial but significant resistance to a challenge with the H5N1 virus (139). In contrast, all mice receiving the vaccine via the intramuscular route succumbed to the heterosubtypic challenge. Ferrets intranasally immunized with the same vaccine survived the H5N1 challenge and displayed reduced nasal viral loads, unlike animals vaccinated via the parenteral route (139).

Numerous pre-clinical studies have proven that i.n. influenza VLP vaccines elicit long-lasting (cross-) protective immune responses (140–142). For example, long-lived IgA/IgG ASCs and memory CD4^+^/CD8^+^ T cells were detected in the bone marrow and in the spleen of mice immunized with H1N1 (HA/M1) VLPs, respectively (140). All mice survived an i.n. homologous or heterovariant challenge up to 5 months post-immunization (140). Also, a recent study showed that the i.n. administration of a mixture of (HA/M1) VLPs individually displaying H1, H3, H5, and H7 HA epitopes significantly protected adult mice against an i.n. homologous (H1N1 or H7N1 virus), heterovariant (H5N1 or H7N9 virus), or heterosubtypic (H2N1, H7N1, H10N1, or H11N1 virus) challenge (142). The VLP cocktail protected mice against a challenge with the H7N9 or H10N1 virus until 6 months post-vaccination. Passive serum transfer experiments suggested an involvement of anti-HA Abs in the resistance against homologous or heterovariant challenge; however the correlates of protection against the heterosubtypic challenge remain unknown (142). Interestingly, a partial protection was also observed in aged mice against the H10N1 challenge (142).

Other VLP platforms presenting conserved IAV epitopes (M2e or epitopes located in HA2) have been successfully tested in pre-clinical tests with the hepatitis B virus core protein (73, 143, 144), the coat protein of bacteriophage Qβ (Qβ-VLPs) (145), the protrusion domain of the capsid protein of norovirus (P-VLPs) (138, 146) or the NP of respiratory syncytial virus (N_RSV_-VLPs) (147) as VLP scaffold.

Experimental studies in mice have allowed a better understanding of the adjuvant mechanisms of Qβ-VLPs at the mucosal surfaces (148, 149). During the self-assembly process, Qβ-VLPs are packaged with RNA derived from *E. coli* which typically mediate TLR3/7 signals. A first study concluded that Qβ-VLPs applied via the i.n. route were captured by alveolar macrophages and lung DCs, transported to the DLN and subsequent mucosal IgA responses required macrophage/DC activation via TLR7 stimulation (148). A second study established that Qβ-VLPs administered intranasally could also be taken up by the lung B cells, transported to the spleen and delivered into B cell follicles (149). In accordance with these results, mice intranasally immunized with Qβ-VLPs incorporating M2e epitopes developed significant mucosal and serum M2e-specific humoral responses, and all animals survived a subsequent i.n. lethal H1N1 challenge (145). Besides Qβ-VLPs, P-VLPs are also efficient mucosal delivery platforms for M2e epitopes in mice (138) and in chickens (146). Chickens intranasally immunized with P-VLPs carrying multiple repetitions of an avian M2e consensus sequence showed a significant reduction in virus shedding from the trachea and the cloaca after an i.n. challenge with avian H5N2 or H7N2 viruses (146). However, no anti-M2e Ab were detected in the serum or in the nasal washes and the protective immune mechanisms remain unknown. Finally, in our research group, we have demonstrated that mice intranasally immunized with three repetitions of M2e sequence from a H1N1 virus exposed at the surface of N_RSV_-VLPs developed strong local and systemic M2e-specific Ab responses and were successfully protected against an i.n. homologous challenge (147). Only the i.n. route generated mucosal IgA titers and led to the protection of animals (147).

Although most of the aforementioned influenza VLPs trigger potent host immune reactions by themselves, several studies have indicated that the incorporation of immunostimulating molecules such as enterotoxins (64, 73), PRR ligands (78, 79, 81, 82, 150) or cytokines/chemokines (151), either admixed or anchored at the surface of VLPs, enhances the magnitude, the duration and/or the breadth of the protective immune responses.

Clinical trials have attested the safety and the efficacy of influenza VLPs in parenteral vaccination (136). The promising results obtained in mucosal vaccination during experimental animal studies support the rationale for developing influenza VLPs for human mucosal vaccination as well.

### Organic Polymers

#### Chitosan

Chitosan are natural cationic polysaccharides composed of glucosamine and *N*-acetylglucosamine resulting from the partial deacetylation of chitin (152). Diverse forms of chitosan are available with various degrees of acetylation, molecular weight and chemical modifications affecting their solubility, biocompatibility, biodegradation and immunogenicity (152). Chitosan polymers and their derivatives have been widely used as mucosal adjuvants under different formulations such as solutions, powders, micro/nanoparticles and gels (152). Because of electrostatic interactions of positively charged chitosan with the negatively charged components of the mucus, chitosan polymers exhibit strong mucoadhesive properties. In addition, chitosan-based adjuvants enhance the permeability of the epithelial barrier by inducing a reversible opening of tight junctions, and consequently promote the interactions of the co-administered Ag(s) with the MALT. Finally, chitosan polymers directly stimulate the activation and the maturation of APCs including DCs via TLR4-, NLR family pyrin domain containing 3 (NLRP3)-, and/or stimulator of interferon genes (STING)-dependent pathway (152, 153).

Chitosan-based adjuvants have successfully improved the immunogenicity and the protective potential of i.n. influenza split vaccines in pre-clinical tests (154–157). For example, *N*-trimethyl-derivatives of chitosan (TMC) boosted the immunogenicity of H1N1 split vaccine reflected by increased serum IgG and HAI Ab titers and mucosal IgA titers (154). An optimal immune response was obtained when influenza Ags were conjugated on TMC nanoparticles instead of being encapsulated into the TMC nanoparticles (154). In another study, a thermo-sensitive chitosan-based hydrogel (a solution of *N*-[(2-hydroxy-3-trimethylammonium) propyl] chitosan chloride (HTCC) plus α,β-glycerophosphate) formulated with H5N1 split vaccine extended the retention time of influenza Ags in the nasal cavity of mice (156). This could be due to the mucoadhesive properties of HTCC and/or the reduction of the ciliary beating frequency caused by the overlaying of epithelial surface with the semi-solid hydrogel solution (156). In addition, the adjuvant efficiently increased the intercellular penetration of influenza Ags through the mucosa (156). Consequently, mice receiving the hydrogel-adjuvanted vaccine presented a boost in influenza-specific serum and mucosal Ab titers as well as in cell-mediated immune responses, including an increase in type 1/type 2 cellular responses in the spleen and in the frequency of memory CD8^+^ T cells in the NALT (156). TMC admixed with H5N1 split vaccine also boosted serum Ab titers reactive against the vaccine strain and H5N1 heterovariant viruses in ferrets and the adjuvanted vaccine protected animals against a subsequent intratracheal homologous challenge (157).

Pre-clinical experiments in mice and chickens have also revealed that chitosan polymers are efficient mucosal adjuvants for DNA (158) or protein influenza subunit vaccines either admixed with influenza Ags (144, 159) or as coating agents on the surface of polyamines (69, 160) or polyesters (161, 162) nanoparticles containing influenza Ags (see sections Chitosan and Polyamine polymers). Chitosan-containing lipid nanoparticles complexed with mRNA encoding H5 HA or NP proteins also boosted the immunogenicity of IAV Ags after subcutaneous immunization in mice and rabbits (163). These experiments have to be performed in the context of a mucosal vaccination.

Clinical studies have proven the safety and the adjuvant potential of chitosan for various i.n. vaccines including IIV vaccines, but further investigations are needed (164, 165).

#### Polyamine Polymers

Poly (γ-glutamic acid) (γ-PGA) polymers are anionic biodegradable bacterial polymers with mucoadhesive properties due to the hydrogen bonding between the carboxylate group of γ-PGA and the hydroxyl group of the mucus glycoproteins (166). In addition, γ-PGA nanoparticles are efficiently taken up by APCs and they enhance the activation, the maturation and the functions of APCs (167).

Experiments in mice have demonstrated that γ-PGA polymers are potent mucosal adjuvants/carriers for split or subunit influenza vaccines (69, 160, 167, 168). For example, a first study established that the supplementation of an i.n. H1N1 split vaccine with γ-PGA nanoparticles enhanced serum and mucosal neutralizing Ab responses and type 1/type 2-cell mediated immune responses in the spleen (168). These responses were specifically directed against the vaccine strain or a heterovariant virus (168). Mice immunized with the adjuvanted vaccine were fully resistant to i.n. homologous or heterovariant challenge (168). In a second study, the incorporation of consM2_H1N1/H5N1/H9N2_-HA2_H5N1(15−137)_ into a formulation consisting of γ-PGA polymers associated with MPLA and QS21 (a specific fraction of QuilA saponins) generated higher and more persistent HA2/M2-specific immune responses than a free consM2_H1N1/H5N1/H9N2_-HA2_H5N1(15−137)_ fusion protein vaccine (167). The adjuvanted vaccine conferred a full and/or long-lasting protection to mice against an i.n. challenge with different subtypes of IAV including H5N1, H1N1, H5N2, or H9N2 viruses (167). Notably, the adjuvant effect of the γ-PGA/MPLA/QS21 formulation was similar as CT (167).

Polyethyleneimine (PEI) polymers are a family of synthetic cationic molecules widely used as gene transfer agents because of their ability to complex, condense and protect nucleic acids against enzymatic degradation, to promote transfection of cells and to facilitate endosomal escape of nucleic acids (166). *In vivo* bioluminescence imaging of luciferase performed in mice after the i.n. administration of luciferase-encoding DNA formulated with PEI or unformulated DNA revealed that PEI significantly improved the efficiency of gene transfer in the RT (169). Accordingly, PEI efficiently boosted the magnitude, the breadth and the protective potential of influenza DNA vaccines administered via the i.n. route in mice (169). In particular, the combination of PEI with a DNA vaccine encoding a HA derived from a H5N1 virus increased the serum and mucosal Ab responses against the vaccine strain (169). Furthermore, the animals presented higher frequency of HA-specific memory IFN-γ-secreting CD4^+^ and CD8^+^ T cells in the lungs and spleen than mice vaccinated with the naked DNA (169). In accordance with these observations, mice immunized with the adjuvanted vaccine were more resistant to an i.n. homologous challenge (169). Finally, the PEI-complexed vaccine generated cross-reactive immune responses and conferred partial cross-protection to mice against an i.n. H5N1 heterovariant challenge (169). A recent study has demonstrated that PEI conjugated with β-cyclodextrin (a cyclic polysaccharide) is an excellent platform material for i.n. mRNA vaccination in mice (170). *In vivo* imaging showed that the complexation of anionic mRNA encoding the envelope glycoprotein gp120 of HIV with the PEI-cyclodextrin polymer prolonged the nasal residence time of the mRNA and increased uptake of the mRNA by nasal epithelial cells (170). In addition, *in vitro* observations suggested that the polymer facilitated the i.n. delivery of the mRNA cargo through a paracellular route by reversibly opening tight junctions while preserving the integrity of the epithelial barrier (170). Consequently, this polymeric delivery system boosted the gp120-specific serum and mucosal humoral and cellular responses (170). In mice, PEI is a potent adjuvant for subcutaneous or intramuscular mRNA vaccines encoding IAV HA or NP Ags (171, 172). However, the ability of PEI to transport IAV mRNA vaccines through the mucosal route awaits further investigation.

Likewise, PEI polymers are potent mucosal adjuvants for influenza WIV and protein subunit vaccines in mice and chickens, with similar potency as bacterial-derived adjuvants (84, 173). *In vitro* analysis showed that the combination of PEI with H9N2 WIV vaccine increased the adhesion of the viral Ags to Calu-3 epithelial cells (173). In addition PEI enhanced the cellular uptake and endosomal escape of the viral Ags in murine bone marrow-derived DCs, and stimulated the maturation of the cells (173). Accordingly, mice intranasally immunized with the PEI-complexed WIV vaccine developed higher local and systemic influenza-specific immune responses than mice immunized with the uncomplexed WIV vaccine (173). Finally, a recent study demonstrated (172, 173) that chickens immunized with a portion of the HA1 domain of a H7N9 virus formulated with PEI developed higher serum, nasal and lung anti-HA Ab titers than animals immunized with the unadjuvanted formulation (84). The birds also showed reduced viral loads in the cloaca and throat after an i.n. homologous challenge in comparison with the birds of the unadjuvanted group (84).

Because of safety issues related to the non-biodegradable nature of PEI and to the positive charge of these polymers (which can interact electrostatically with cellular anionic macromolecules and interfere with normal cellular functions), less toxic PEI derivatives have been developed such as deacylated PEI. These modified PEI polymers retain adjuvant properties toward HA DNA vaccines in mice (174).

#### Polyesters

PLGA, a copolymer of poly (D, L-lactide-co-glycolide) approved for clinical use, has been widely exploited as a nanoparticle delivery system because of its biocompatibility, biodegradability, safety, and controlled release properties (166). Hydrophilic polymeric materials can be added on the surface of PLGA nanoparticles to increase the stability and the transfer of the antigenic payload across mucosal surfaces. These stabilizers are usually polyethylene glycol, polyvinyl alcohol or chitosan (175). Experimental studies in animals have demonstrated that PLGA nanoparticles are good mucosal adjuvants for i.n. IAV WIV vaccines (162, 176). A first study showed that chickens immunized with an aerosol vaccine composed of H9N2 WIV admixed with polyvinyl alcohol-modified PLGA nanoparticles incorporating PEI-CpG-ODN complexes generated higher virus-specific serum IgY and HAI Ab titers and mucosal IgA titers than birds immunized with the non-adjuvanted WIV vaccine (176). A second study concluded that chickens immunized via i.n. and intraocular routes with polyvinyl alcohol/chitosan-modified PLGA nanoparticles encapsulating a H4N6 WIV with PEI-CpG-ODN complexes developed higher influenza-specific serum and mucosal Ab responses than chickens of the unadjuvanted group (162).

Poly-(ε-caprolactone), another biodegradable and biocompatible polyester polymer, was also described as a potent carrier system for mucosal vaccines against IAV in pre-clinical tests (161). Notably, mice intranasally vaccinated with chitosan-coated poly-(ε-caprolactone) nanoparticles incorporating a recombinant HA derived from a H1N1 virus exhibited higher HA-specific serum and mucosal Ab titers and systemic type 1/type 2-cell-mediated immune responses than mice vaccinated with uncoated nanoparticles or with the unadjuvanted recombinant HA (161).

#### Other Organic Polymers

Poly (*N*-vinylacetamide-*co*-acrylic acid) (PNVA-co-AA) bearing D-octaarginine are biocompatible cationic oligopeptides which enhance permeation of co-mixed Ag(s) through mucosal barriers (177). The co-administered Ag(s) are suggested to be taken up into cells via macropinocytosis through the biorecognition of the peptidyl branches in the polymer backbone, while the polymer remains on the cell membrane (177). The i.n. co-administration of H1N1 WIV vaccine with D-octaarginine-linked PNVA-co-AA in mice boosted the HA-specific nasal IgA titers which cross-reacted *in vitro* with recombinant HAs from H1N1 heterovariant and H3N2 or H5N1 heterosubtypic viruses (178, 179). Mice vaccinated with the adjuvanted preparation showed a better resistance to an i.n. homologous challenge than mice immunized with H1N1 WIV alone (178, 179). The cross-protective potential of such polymers remains to be evaluated *in vivo*.

### Inorganic Nanoparticles

During the last years, gold nanoparticles are appearing increasingly attractive (mucosal) vaccine delivery vehicles (180). As immunologically inert molecules, they do not induce competing carrier-specific immune responses. Also, they can be chemically synthesized with tight control over the nanoparticle size and are easily functionalized with vaccine epitopes/immunopotentiators (180). A series of recent publications has demonstrated that gold nanoparticles are potent mucosal delivery platforms for influenza Ags in mice (81, 82, 181, 182). By designing dual-linker nanoparticles, a research group grafted a recombinant trimeric HA derived from a H3N2 virus and a recombinant flagellin monomer on gold nanoparticles by click chemistry and metal-chelating reactions with high conjugation efficiency (81, 82). The conjugation of the vaccine epitopes on the nanoparticles increased the uptake of influenza Ags by DCs and stimulated the activation, maturation and function of DCs *in vitro* (82). With respect to these observations, the vaccine formulation intranasally administered to mice generated stronger influenza-specific immune responses than a vaccine composed of free trimeric HA mixed with flagellin (81). Notably, mice immunized with the nanoparticles showed elevated serum IgG and HAI Ab titers, higher IgG and IgA titers in the URT and LRT and higher frequency of long-lived IgG/IgA ASCs in the spleen (81). In addition, animals exhibited higher influenza-specific systemic and local cell-mediated immune responses. In particular, mice showed a boost in type 1/type 2/type 17-cell-mediated immune responses and in the frequency of CD8^+^ CTLs in the spleen, an increase in type 1-biased cellular responses in the NALT and higher frequency of IFN-γ-expressing CD4^+^/CD8^+^ T cells in the DLN (81). All mice survived a lethal i.n. homologous challenge and showed reduced lung viral loads in contrast to animals receiving the vaccine formulation uncoupled to the nanoparticles (81). The attachment of a human M2e consensus sequence to gold nanoparticles also significantly strengthened the immunogenicity and the protective potential of the M2e epitopes in mice (181, 182). Hence, these nanocarriers are interesting mucosal delivery platforms for broadly-protective vaccines against IAV.

### Nucleotide-Based Adjuvants

Polyinosine-polycytidylic acid [poly (I:C)] is a synthetic analog for double-stranded RNA that mimics viral RNA and activates various APCs in a TLR3/Toll or interleukin (IL)-1 receptor domain-containing adaptor-inducing interferon-β (TRIF)-dependent pathway (61). Numerous studies have demonstrated that the incorporation of poly (I:C) into IAV WIV, split or subunit vaccines enhanced the magnitude and/or the breadth of the specific immune in mice and chickens (183–186).

A recent study showed that the i.n. administration of poly (I:C) with H1N1 split vaccine in mice boosted serum IgG and mucosal IgA Ab responses in a TLR3/TRIF-dependent pathway (183). The adjuvant also increased the frequency of virus-specific IFN-γ-secreting CD4^+^ and CD8^+^ T cells in the spleen (183). The mucosal adjuvanticity of poly (I:C) relied on its ability to stimulate the activation and the maturation of CD103^+^ DCs located in the NALT, which were crucially involved in the generation of local humoral and T cell-mediated immune responses (183). In particular, the addition of poly (I:C) to the vaccine formulation enhanced the frequency of GC B cells and follicular helper T cells in the NALT and the local expression of various cytokines involved in IgA class switching (183). Mice immunized with the adjuvanted vaccine were fully protected against an i.n. H1N1 heterovariant challenge in contrast to TRIF^−/−^ mice or mice immunized with the unadjuvanted vaccine (183).

Poly (I:C) was also tested in a HA2-directed vaccine strategy in mice (185). The methodology consisted of sequential immunizations with different chimeric HA molecules which have the same H1 HA2 but different non-H1 HA1s. The adjuvant boosted the induction of HA2-specific serum IgG and nasal IgA directed against the HA vaccine strain (185). In addition the adjuvant enhanced the generation of serum Abs which cross-reacted with HA molecules from H1N1 heterovariant and H5N1 and H2N2 heterosubtypic viruses (185). The adjuvanted vaccine fully protected mice against i.n. homologous and H1N1 heterovariant challenges (185).

We can mention that poly (I:C) is also an interesting mucosal adjuvant for influenza vaccines in chickens (186). The i.n. co-administration of poly (I:C) with H5N1 IIV vaccine enhanced influenza-specific serum IgG and HAI Ab titers and IgA titers in the nose and in the trachea of birds (186).

Finally, poly (I:C_12_U), an analog of poly (I:C) exhibiting a safer profile, gave promising results in pre-clinical (187) and clinical trials (188). In particular, the i.n. immunization of healthy humans with a seasonal LAIV vaccine in association with poly (I:C_12_U) induced the generation of nasal IgA which reacted with homologous viruses as well as H5N1, H7N9, and H7N3 HPAIV with pandemic potential for humans (188).

Another class of nucleotide-based adjuvant, CDN, exhibited comparable efficacy to poly (I:C) in enhancing the immunogenicity of mucosal IAV split or subunit vaccines in mice (114, 189–192). CDN are bacterial second-messenger molecules detected by the innate immune system via various sensors including STING (189). The inclusion of 2′,3′-cyclic-guanosine monophosphate-adenosine monophosphate (cGAMP) in i.n. H1N1 split vaccine stimulated the activation of innate and adaptive immunity in the NALT, the spleen and the DLN, and promoted GC formation in the NALT in a STING-dependent pathway (189). It resulted in an increase in specific serum and mucosal humoral responses and in systemic type 1/type 2/type 17 cellular immune responses, including higher frequency of IFN-γ-producing CD4^+^ and CD8^+^ T cells in the spleen (189). Other studies demonstrated that the addition of CDN to recombinant H5N1 HA or H1N1 NP subunit vaccines enhanced the magnitude and/or the breadth of the humoral and cellular anti-IAV immune responses in mice (190, 192).

CpG-ODN are TLR9/TLR21 agonists which activate various APCs and commonly generate pro-inflammatory and Th1-biaised immune milieu (193). These compounds have thus been widely exploited as vaccine enhancers in numerous pre-clinical and human clinical trials (193). Several studies in mice and chickens have demonstrated that CpG-ODN alone or in combination with other immunopotentiators/delivery systems are suitable mucosal adjuvants for IAV WIV, split or subunit vaccines (64, 83, 99, 176, 181, 182, 186, 194–197). By using an *in vitro* DCs:Calu-3 cells co-culture model and by doing i.n. instillation in mice *in vivo*, a recent study proposed a mechanism for the mucosal adjuvanticity of CpG-ODN toward a H9N2 WIV vaccine. The authors suggested that CpG-ODN stimulated the secretion of the chemokine (C-C motif) ligand 20 (CCL20) by epithelial cells in a TLR9-dependent pathway, which enhanced DC recruitment to the nasal epithelium and promoted the formation of transepithelial dendrites involved in the capture of luminal influenza Ags (197). Clinical trials have proven the safety and immunogenicity of CpG-ODN-adjuvanted IIV vaccines when administered via the parenteral route (85). In view of the very promising results in animal models, the efficacy of CpG-ODN in mucosal anti-IAV vaccination should be evaluated in humans.

### Cytokines/Chemokines

Cytokines and chemokines are signaling molecules released by a wide range of immune and non-immune cells (198). They are critically involved in host defense against infections by modulating the activation, maturation, differentiation, migration, survival and functions of innate and adaptive immune cells (198). These molecules have been considered as interesting alternatives to pathogen-derived adjuvants for anti-IAV vaccines (198).

Various cytokines such as IL-2, IL-12, IL-1 family cytokines (IL-1α/β, IL-18, and IL-33), IL-23 and type I IFNs are efficient and safe mucosal adjuvants when admixed with IAV WIV, split or subunit vaccines in mice (198, 199). For example, the incorporation of type I IFNs to an i.n. H1N1 split vaccine enhanced the magnitude and the duration of virus-specific serum and mucosal humoral responses and increased the protection of mice against an i.n. homologous challenge (200). The mucosal adjuvanticity of type I IFNs was suggested to be related to the ability of the cytokines to stimulate the uptake of co-administered Ags by phagocytes of the nasal mucosa (200). In another study in mice, the i.n. co-administration of cytokines belonging to the IL-1 family with recombinant HA derived from a H1N1 virus increased the magnitude of serum and mucosal anti-HA Abs titers in serum and in mucosal secretions as well as the frequency of type 1/type 2 cytokine-producing cells and CD8^+^ CTLs in the spleen (201). The adjuvant remarkably enhanced the level of protection of mice against an i.n. heterovariant challenge (201). Mucosal vaccine formulations with cytokines/chemokines anchored into influenza WIV or VLPs have also been successfully tested in mice (151, 202–204). The incorporation of cytokines/chemokines in nanoparticle vaccine formulations could overcome the relatively short half-life of these molecules (198). Recent studies have been focused on CCL28, which is highly expressed by epithelial cells and selectively attracts T and B cell subsets (including IgA ASCs) at mucosal surfaces (151, 203). CCL28 anchored in H3N2 (HA/M1) VLPs showed *in vitro* chemotactic activity toward chemokine (C-C motif) receptor 3 (CCR3)/CCR10-expressing mouse splenocytes and lung cells (203). In mice, the chemokine enhanced the magnitude, the avidity, the functionality and the duration of serum and mucosal humoral responses specifically directed against homologous and heterovariant viruses (151, 203). Accordingly, the adjuvanted vaccine conferred a higher and more persistent protection in animals against an i.n. homologous or heterovariant challenge (151, 203).

Although cytokines/chemokines are promising adjuvants for mucosal anti-IAV vaccines, these molecules may present some drawbacks (198). The pleiotropic effects of these proteins on immune cells may result in unwanted adverse effects. In addition, the adjuvant effects observed in animal models may not be directly translated to humans as it was reported for an i.n. influenza split vaccine formulated with type I IFNs (205).

### Adjuvants and Trained Immunity

Studies listed so far in this review have evaluated the efficacy of mucosal adjuvants on their ability to enhance adaptive immune responses (T and/or B cell responses) specifically directed against influenza Ags (Supplemental Table 1). However, a growing body of evidence suggests that some categories of adjuvants may also induce long-lasting functional state within innate immune cells, resulting in an increase in non-specific host defense against a range of pathogens. This concept has been named “trained immunity” (206). Some studies have indicated that prophylactic mucosal administration of bacterial-derived molecules, in particular enterotoxins, TLR3 and TLR9 ligands, or type I IFNs protected mice against a subsequent IAV challenge (73, 207–209). For example, mice intranasally treated with CT, LT (R192G) or CpG-ODN showed reduced mortality and/or lung viral loads subsequently to a H1N1 challenge 24 h after the last treatment. The improved resistance was associated with higher frequencies of CD4^+^ T cells, B cells and DCs in the airways and the generation of BALT-like structures in the lungs (207). Furthermore, the i.n. administration of chitosan fully protected mice against lethal challenge with H7N9 or diverse H1N1 viruses (210). The protection was associated with infiltration of leukocytes in the bronchoalveolar lavage and an enhanced expression of inflammatory cytokines in the LRT (210). Mice were significantly protected against H7N9 challenge even 10 days after the chitosan administration (210). Whether the protective efficacy of the mucosal IAV vaccine formulations compiled in this review was influenced by trained immunity remains to be determined.

## Conclusions

Vaccination via the i.n. route has the potential to elicit long-lasting and cross-protective humoral and cellular immune responses at the portal of entry of respiratory pathogens. It is thus a suitable strategy to prevent infections caused by highly variable IAV. However, the i.n. vaccine formulations have to be rationally elaborated because the respiratory mucosal surfaces restrain the immunoavailability and the immunogenicity of vaccine Ag(s). Numerous experimental animal studies have demonstrated the promising potential of various adjuvant/delivery systems for the development of i.n. human and veterinary (chickens) vaccines against IAV. They consist of immunopotentiatory molecules, such as bacterial-derived components, synthetic nucleotides and cytokines/chemokines, associated or not with particulate carriers, such as lipid-based particles, VLPs, organic polymers and inorganic nanoparticles. These adjuvant systems boost the efficacy of current IIV vaccines or new subunit vaccine candidates by increasing the magnitude, the persistence and/or the breadth of the host (protective) anti-IAV immunity. Some of these novel mucosal vaccine formulations are safe and immunogenic in early phase clinical trials but they have to overcome several hurdles before reaching the market, including regulatory and economic restrictions and the lack of appropriate correlates of protection. Standardized assays taking into account the protective role of non-HAI Abs and cell-mediated immunity need to be developed to adequately evaluate the efficacy of these new mucosal formulations. Needle-free mucosal vaccines that provide a broad-coverage against IAV and do not require annual re-vaccination are promising alternatives to current IAV vaccines and should increase awareness about the benefits of influenza vaccination among the general public.

## Author Contributions

CCa and CCh have written, prepared, and approved the manuscript for publication.

### Conflict of Interest Statement

The authors declare that the research was conducted in the absence of any commercial or financial relationships that could be construed as a potential conflict of interest.

## Supplementary Material

Supplemental Table 1 Adjuvants/delivery systems tested in intranasal vaccination against IAV infections in pre-clinical and clinical studies.

## Abbreviations

Ab: antibody
Ag: antigen
APC: antigen-presenting cell
ASC: antibody-secreting cell
BALT: bronchus-associated lymphoid tissues
BCR: B cell receptor
CCL: (C-C motif) ligand
CCR: (C-C motif) receptor
CCS: ceramide carbamoyl-spermine
cDC: conventional dendritic cell
c-di-AMP: cyclic-di-adenosine monophosphate
cGAMP: 2’3’-cyclic-guanosine monophosphate-adenosine monophosphate
CDN: cyclic di-nucleotides
CpG-ODN: synthetic oligodeoxynucleotides composed of unmethylated CpG motifs
CT: cholera toxin
CTA1: A1 subunit of the cholera toxin
CTA1-DD: CTA1 fused to a synthetic dimer of the Ig binding D domain (DD) from *Staphylococcus aureus* protein A
CTL: cytotoxic T lymphocyte
DC: dendritic cell
DDAB: dimethyldioctadecylammonium bromide
DLN: draining lymph nodes
DMPC: dimyristoyl phosphatidylcholine
DMPG: dimyristoyl phosphatidylglycerol
DOTAP: dioleoyl-3-trimethylammoniumpropane
DPPC: dipalmitoyl phosphatidylcholine
FAE: follicle-associated epithelium
GC: germinal center
HA: hemagglutinin
HA1: globular head domain of hemagglutinin
HA2: stalk domain of hemagglutinin
HAI: hemagglutination-inhibition
HIV: human immunodeficiency virus
HPAIV: highly pathogenic avian influenza viruses
HTCC: *N*-[(2-hydroxy-3-trimethylammonium) propyl] chitosan chloride
IAV: influenza A viruses
IBV: influenza B virus
IFN: interferon
Ig: immunoglobulins
IIV: inactivated influenza viruses
IL: interleukin
i.n.: intranasal
ISCOM: immune stimulating complex
ISCOMATRIX: immune stimulating complex matrix
LAIV: live-attenuated influenza viruses
LPAIV: low pathogenic avian influenza viruses
LRT: lower respiratory tract
LT: *Escherichia coli* heat-labile toxin
M cells: microfold cells
M1: matrix protein 1
M2(e): (ectodomain of the) matrix protein 2
MALT: mucosa-associated lymphoid tissues
mDC: myeloid dendritic cell
MHC: major histocompatibility complex
moDC: monocyte-derived dendritic cell
MPLA: monophosphoryl lipid A
NA: neuraminidase
NAIP: nucleotide oligomerization domain-like receptor family, apoptosis inhibitory protein
NALT: nasopharynx-associated lymphoid tissues
NK: natural killer
NLR: nucleotide oligomerization domain-like receptor
NLRP3: nucleotide oligomerization domain-like receptor family, pyrin domain containing 3
NP: nucleoprotein
N_RSV_: nucleoprotein of the respiratory syncytial virus
PA: polymerase acidic protein
PAMP: pathogen associated molecular pattern
PB1: polymerase basic protein 1
PB2: polymerase basic protein 2
pDC: plasmacytoid dendritic cell
PEI: polyethyleneimine
PG: phosphatidylglycerol
γ-PGA: poly (γ-glutamic acid)
PLGA, poly (D: L-lactide-co-glycolide)
poly (I:C): polyinosine-polycytidylic acid
PRR: pattern recognition receptor
RT: respiratory tract
SP-C: surfactant protein C
STING: stimulator of interferon genes
Th: T helper
TLR: Toll-like receptor
TMC: *N*-trimethyl-derivatives of chitosan
TRIF: Toll or interleukin-1 receptor domain-containing adaptor-inducing interferon-β
T_RM_: resident memory T cell
URT: upper respiratory tract
VLP: virus-like particle
WIV: whole inactivated viruses.

## References

[1] Rajao DS Perez DR. Universal vaccines and vaccine platforms to protect against influenza viruses in humans and agriculture. Front Microbiol. (2018) 9:123. 10.3389/fmicb.2018.00123 29467737 PMC5808216

[2] Organization WH Influenza (Seasonal) Fact Sheet. (2017). Available online at: https://www.who.int/news-room/fact-sheets/detail/influenza-(seasonal)

[3] Pantin-Jackwood MJ Swayne DE. Pathogenesis and pathobiology of avian influenza virus infection in birds. Rev Sci Tech. (2009) 28:113–36. 10.20506/rst.28.1.1869 19618622

[4] Tamura S Ainai A Suzuki T Kurata T Hasegawa H. Intranasal inactivated influenza vaccines: a reasonable approach to improve the efficacy of influenza vaccine? Jpn J Infect Dis. (2016) 69:165–79. 10.7883/yoken.JJID.2015.560 27212584

[5] Cerutti A Chen K Chorny A. Immunoglobulin responses at the mucosal interface. Annu Rev Immunol. (2011) 29:273–93. 10.1146/annurev-immunol-031210-101317 21219173 PMC3064559

[6] de Geus ED Rebel JM Vervelde L. Induction of respiratory immune responses in the chicken; implications for development of mucosal avian influenza virus vaccines. Vet Q. (2012) 32:75–86. 10.1080/01652176.2012.711956 22862156

[7] Pulendran B Maddur MS. Innate immune sensing and response to influenza. Curr Top Microbiol Immunol. (2015) 386:23–71. 10.1007/82_2014_405 25078919 PMC4346783

[8] Vangeti S Yu M Smed-Sorensen A. Respiratory mononuclear phagocytes in human influenza A virus infection: their role in immune protection and as targets of the virus. Front Immunol. (2018) 9:1521. 10.3389/fimmu.2018.01521 30018617 PMC6037688

[9] Oshansky CM Thomas PG. The human side of influenza. J Leukoc Biol. (2012) 92:83–96. 10.1189/jlb.1011506 22362872 PMC3382310

[10] GeurtsvanKessel CH Willart MA van Rijt LS Muskens F Kool M Baas C Clearance of influenza virus from the lung depends on migratory langerin^+^CD11b^−^ but not plasmacytoid dendritic cells. J Exp Med. (2008) 205:1621–34. 10.1084/jem.20071365 18591406 PMC2442640

[11] Helft J Manicassamy B Guermonprez P Hashimoto D Silvin A Agudo J. Cross-presenting CD103^+^ dendritic cells are protected from influenza virus infection. J Clin Invest. (2012) 122:4037–47. 10.1172/JCI60659 23041628 PMC3484433

[12] Ho AW Prabhu N Betts RJ Ge MQ Dai X Hutchinson PE. Lung CD103^+^ dendritic cells efficiently transport influenza virus to the lymph node and load viral antigen onto MHC class I for presentation to CD8 T cells. J Immunol. (2011) 187:6011–21. 10.4049/jimmunol.1100987 22043017

[13] Kim TS Braciale TJ. Respiratory dendritic cell subsets differ in their capacity to support the induction of virus-specific cytotoxic CD8^+^ T cell responses. PLoS ONE. (2009) 4:e4204. 10.1371/journal.pone.0004204 19145246 PMC2615220

[14] Ballesteros-Tato A Leon B Lund FE Randall TD. Temporal changes in dendritic cell subsets, cross-priming and costimulation via CD70 control CD8^+^ T cell responses to influenza. Nat Immunol. (2010) 11:216–24. 10.1038/ni.1838 20098442 PMC2822886

[15] Belz GT Smith CM Kleinert L Reading P Brooks A Shortman K. Distinct migrating and nonmigrating dendritic cell populations are involved in MHC class I-restricted antigen presentation after lung infection with virus. Proc Natl Acad Sci USA. (2004) 101:8670–5. 10.1073/pnas.0402644101 15163797 PMC423253

[16] Aldridge JR Jr Moseley CE Boltz DA Negovetich NJ Reynolds C Franks J. TNF/iNOS-producing dendritic cells are the necessary evil of lethal influenza virus infection. Proc Natl Acad Sci USA. (2009) 106:5306–11. 10.1073/pnas.0900655106 19279209 PMC2664048

[17] Jego G Palucka AK Blanck JP Chalouni C Pascual V Banchereau J. Plasmacytoid dendritic cells induce plasma cell differentiation through type I interferon and interleukin 6. Immunity. (2003) 19:225–34. 10.1016/S1074-7613(03)00208-5 12932356

[18] Hemann EA Sjaastad LE Langlois RA Legge KL. Plasmacytoid dendritic cells require direct infection to sustain the pulmonary influenza A virus-specific CD8 T Cell Response. J Virol. (2015) 90:2830–7. 10.1128/JVI.02546-15 26719269 PMC4810669

[19] McGill J Van Rooijen N Legge KL. Protective influenza-specific CD8 T cell responses require interactions with dendritic cells in the lungs. J Exp Med. (2008) 205:1635–46. 10.1084/jem.20080314 18591411 PMC2442641

[20] Cole SL Ho LP. Contribution of innate immune cells to pathogenesis of severe influenza virus infection. Clin Sci. (2017) 131:269–83. 10.1042/CS20160484 28108632

[21] Lawrence CW Braciale TJ. Activation, differentiation, and migration of naive virus-specific CD8^+^ T cells during pulmonary influenza virus infection. J Immunol. (2004) 173:1209–18. 10.4049/jimmunol.173.2.1209 15240712

[22] Roman E Miller E Harmsen A Wiley J Von Andrian UH Huston G. CD4 effector T cell subsets in the response to influenza: heterogeneity, migration, and function. J Exp Med. (2002) 196:957–68. 10.1084/jem.20021052 12370257 PMC2194021

[23] Topham DJ Tripp RA Doherty PC. CD8^+^ T cells clear influenza virus by perforin or Fas-dependent processes. J Immunol. (1997) 159:5197–200. 9548456

[24] Brincks EL Katewa A Kucaba TA Griffith TS Legge KL. CD8 T cells utilize TRAIL to control influenza virus infection. J Immunol. (2008) 181:4918–25. 10.4049/jimmunol.181.7.4918 18802095 PMC2610351

[25] Hua L Yao S Pham D Jiang L Wright J Sawant D. Cytokine-dependent induction of CD4^+^ T cells with cytotoxic potential during influenza virus infection. J Virol. (2013) 87:11884–93. 10.1128/JVI.01461-13 23986597 PMC3807312

[26] Brown DM Lee S Garcia-Hernandez Mde L Swain SL. Multifunctional CD4 cells expressing gamma interferon and perforin mediate protection against lethal influenza virus infection. J Virol. (2012) 86:6792–803. 10.1128/JVI.07172-11 22491469 PMC3393557

[27] Brown DM Dilzer AM Meents DL Swain SL. CD4 T cell-mediated protection from lethal influenza: perforin and antibody-mediated mechanisms give a one-two punch. J Immunol. (2006) 177:2888–98. 10.4049/jimmunol.177.5.2888 16920924

[28] Wilkinson TM Li CK Chui CS Huang AK Perkins M Liebner JC. Preexisting influenza-specific CD4^+^ T cells correlate with disease protection against influenza challenge in humans. Nat Med. (2012) 18:274–80. 10.1038/nm.2612 22286307

[29] Sridhar S Begom S Bermingham A Hoschler K Adamson W Carman W. Cellular immune correlates of protection against symptomatic pandemic influenza. Nat Med. (2013) 19:1305–12. 10.1038/nm.3350 24056771

[30] McMichael AJ Gotch FM Noble GR Beare PA. Cytotoxic T-cell immunity to influenza. N Engl J Med. (1983) 309:13–7. 10.1056/NEJM198307073090103 6602294

[31] Kumar A Meldgaard TS Bertholet S. Novel platforms for the development of a universal influenza vaccine. Front Immunol. (2018) 9:600. 10.3389/fimmu.2018.00600 29628926 PMC5877485

[32] Sant AJ DiPiazza AT Nayak JL Rattan A Richards KA. CD4 T cells in protection from influenza virus: viral antigen specificity and functional potential. Immunol Rev. (2018) 284:91–105. 10.1111/imr.12662 29944766 PMC6070306

[33] Sant AJ Richards KA Nayak J. Distinct and complementary roles of CD4 T cells in protective immunity to influenza virus. Curr Opin Immunol. (2018) 53:13–21. 10.1016/j.coi.2018.03.019 29621639 PMC6141328

[34] Takahashi Y Onodera T Adachi Y Ato M. Adaptive B cell responses to influenza virus infection in the lung. Viral Immunol. (2017) 30:431–7. 10.1089/vim.2017.0025 28661720

[35] Boyden AW Legge KL Waldschmidt TJ. Pulmonary infection with influenza A virus induces site-specific germinal center and T follicular helper cell responses. PLoS ONE. (2012) 7:e40733. 10.1371/journal.pone.0040733 22792401 PMC3394713

[36] Joo HM He Y Sangster MY. Broad dispersion and lung localization of virus-specific memory B cells induced by influenza pneumonia. Proc Natl Acad Sci USA. (2008) 105:3485–90. 10.1073/pnas.0800003105 18299574 PMC2265167

[37] Hasegawa H van Reit E Kida H. Mucosal immunization and adjuvants. Curr Top Microbiol Immunol. (2015) 386:371–80. 10.1007/82_2014_402 25015787 PMC7120566

[38] He W Mullarkey CE Duty JA Moran TM Palese P Miller MS. Broadly neutralizing anti-influenza virus antibodies: enhancement of neutralizing potency in polyclonal mixtures and IgA backbones. J Virol. (2015) 89:3610–8. 10.1128/JVI.03099-14 25589655 PMC4403395

[39] Renegar KB Small PA Jr Boykins LG Wright PF. Role of IgA versus IgG in the control of influenza viral infection in the murine respiratory tract. J Immunol. (2004) 173:1978–86. 10.4049/jimmunol.173.3.1978 15265932

[40] Gould VMW Francis JN Anderson KJ Georges B Cope AV Tregoning JS. Nasal IgA provides protection against human influenza challenge in volunteers with low serum influenza antibody titre. Front Microbiol. (2017) 8:900. 10.3389/fmicb.2017.00900 28567036 PMC5434144

[41] Krammer F. The human antibody response to influenza A virus infection and vaccination. Nat Rev Immunol. (2019). 19:383–97 10.1038/s41577-019-0143-6 30837674

[42] Corti D Cameroni E Guarino B Kallewaard NL Zhu Q Lanzavecchia A. Tackling influenza with broadly neutralizing antibodies. Curr Opin Virol. (2017) 24:60–9. 10.1016/j.coviro.2017.03.002 28527859 PMC7102826

[43] Jegaskanda S. The potential role of Fc-receptor functions in the development of a universal influenza vaccine. Vaccines. (2018) 6:27 10.3390/vaccines6020027 29772781 PMC6027188

[44] Jegaskanda S Vanderven HA Wheatley AK Kent SJ. Fc or not Fc; that is the question: antibody Fc-receptor interactions are key to universal influenza vaccine design. Hum Vaccin Immunother. (2017) 13:1–9. 10.1080/21645515.2017.1290018 28332900 PMC5489290

[45] Vanderven HA Jegaskanda S Wheatley AK Kent SJ. Antibody-dependent cellular cytotoxicity and influenza virus. Curr Opin Virol. (2017) 22:89–96. 10.1016/j.coviro.2016.12.002 28088123

[46] Wu T Hu Y Lee YT Bouchard KR Benechet A Khanna K. Lung-resident memory CD8 T cells. (T_RM_) are indispensable for optimal cross-protection against pulmonary virus infection. J Leukoc Biol. (2014) 95:215–24. 10.1189/jlb.0313180 24006506 PMC3896663

[47] Slutter B Van Braeckel-Budimir N Abboud G Varga SM Salek-Ardakani S Harty JT. Dynamics of influenza-induced lung-resident memory T cells underlie waning heterosubtypic immunity. Sci Immunol. (2017) 2:eaag2031 10.1126/sciimmunol.aag2031 28783666 PMC5590757

[48] Pizzolla A Nguyen THO Smith JM Brooks AG Kedzieska K Heath WR. Resident memory CD8^+^ T cells in the upper respiratory tract prevent pulmonary influenza virus infection. Sci Immunol. (2017) 2:eaam6970 10.1126/sciimmunol.aam6970 28783656

[49] Turner DL Bickham KL Thome JJ Kim CY D'Ovidio F Wherry EJ. Lung niches for the generation and maintenance of tissue-resident memory T cells. Mucosal Immunol. (2014) 7:501–10. 10.1038/mi.2013.67 24064670 PMC3965651

[50] Teijaro JR Turner D Pham Q Wherry EJ Lefrancois L Farber DL. Cutting edge: tissue-retentive lung memory CD4 T cells mediate optimal protection to respiratory virus infection. J Immunol. (2011) 187:5510–4. 10.4049/jimmunol.1102243 22058417 PMC3221837

[51] Sridhar S. Heterosubtypic T-Cell immunity to influenza in humans: challenges for universal T-cell influenza vaccines. Front Immunol. (2016) 7:195. 10.3389/fimmu.2016.00195 27242800 PMC4871858

[52] Adachi Y Onodera T Yamada Y Daio R Tsuiji M Inoue T. Distinct germinal center selection at local sites shapes memory B cell response to viral escape. J Exp Med. (2015) 212:1709–23. 10.1084/jem.20142284 26324444 PMC4577849

[53] Zens KD Chen JK Farber DL. Vaccine-generated lung tissue-resident memory T cells provide heterosubtypic protection to influenza infection. JCI Insight. (2016) 1:e85832 10.1172/jci.insight.85832 27468427 PMC4959801

[54] Suarez DL Pantin-Jackwood MJ. Recombinant viral-vectored vaccines for the control of avian influenza in poultry. Vet Microbiol. (2017) 206:144–51. 10.1016/j.vetmic.2016.11.025 27916319

[55] de Vries RD Rimmelzwaan GF. Viral vector-based influenza vaccines. Hum Vaccin Immunother. (2016) 12:2881–901. 10.1080/21645515.2016.1210729 27455345 PMC5137548

[56] Lee LYY Izzard L Hurt AC. A review of DNA vaccines against influenza. Front Immunol. (2018) 9:1568. 10.3389/fimmu.2018.01568 30038621 PMC6046547

[57] Pardi N Hogan MJ Porter FW Weissman D. mRNA vaccines-a new era in vaccinology. Nat Rev Drug Discov. (2018) 17:261–79. 10.1038/nrd.2017.243 29326426 PMC5906799

[58] Zhang C Maruggi G Shan H Li J. Advances in mRNA vaccines for infectious diseases. Front Immunol. (2019) 10:594. 10.3389/fimmu.2019.00594 30972078 PMC6446947

[59] Awate S Babiuk LA Mutwiri G. Mechanisms of action of adjuvants. Front Immunol. (2013) 4:114. 10.3389/fimmu.2013.00114 23720661 PMC3655441

[60] Aoshi T. Modes of action for mucosal vaccine adjuvants. Viral Immunol. (2017) 30:463–70. 10.1089/vim.2017.0026 28436755 PMC5512297

[61] Apostolico Jde S Lunardelli VA Coirada FC Boscardin SB Rosa DS. Adjuvants: classification, modus operandi, and licensing. J Immunol Res. (2016) 2016:1459394. 10.1155/2016/1459394 27274998 PMC4870346

[62] Woodrow KA Bennett KM Lo DD. Mucosal vaccine design and delivery. Annu Rev Biomed Eng. (2012) 14:17–46. 10.1146/annurev-bioeng-071811-150054 22524387

[63] Lycke N Lebrero-Fernandez C. ADP-ribosylating enterotoxins as vaccine adjuvants. Curr Opin Pharmacol. (2018) 41:42–51. 10.1016/j.coph.2018.03.015 29702466

[64] Quan FS Ko EJ Kwon YM Joo KH Compans RW Kang SM. Mucosal adjuvants for influenza virus-like particle vaccine. Viral Immunol. (2013) 26:385–95. 10.1089/vim.2013.0013 24236855 PMC3868302

[65] Quan FS Compans RW Nguyen HH Kang SM. Induction of heterosubtypic immunity to influenza virus by intranasal immunization. J Virol. (2008) 82:1350–9. 10.1128/JVI.01615-07 18032492 PMC2224423

[66] Tumpey TM Renshaw M Clements JD Katz JM. Mucosal delivery of inactivated influenza vaccine induces B-cell-dependent heterosubtypic cross-protection against lethal influenza A H5N1 virus infection. J Virol. (2001) 75:5141–50. 10.1128/JVI.75.11.5141-5150.2001 11333895 PMC114919

[67] Quan FS Yoo DG Song JM Clements JD Compans RW Kang SM. Kinetics of immune responses to influenza virus-like particles and dose-dependence of protection with a single vaccination. J Virol. (2009) 83:4489–97. 10.1128/JVI.02035-08 19211762 PMC2668456

[68] Zhang Y Yu X Hou L Chen J Li P Qiao X. CTA1: Purified and display onto gram-positive enhancer matrix (GEM) particles as mucosal adjuvant. Protein Expr Purif. (2018) 141:19–24. 10.1016/j.pep.2017.08.010 28866467

[69] Chowdhury MYE Kim TH Uddin MB Kim JH Hewawaduge CY Ferdowshi Z. Mucosal vaccination of conserved sM2, HA2 and cholera toxin subunit A1 (CTA1) fusion protein with poly gamma-glutamate/chitosan nanoparticles (PC NPs) induces protection against divergent influenza subtypes. Vet Microbiol. (2017) 201:240–51. 10.1016/j.vetmic.2017.01.020 28284616

[70] Helgeby A Robson NC Donachie AM Beackock-Sharp H Lovgren K Schon K. The combined CTA1-DD/ISCOM adjuvant vector promotes priming of mucosal and systemic immunity to incorporated antigens by specific targeting of B cells. J Immunol. (2006) 176:3697–706. 10.4049/jimmunol.176.6.3697 16517738

[71] Eliasson DG El Bakkouri K Schon K Ramne A Festjens E Lowenadler B. CTA1-M2e-DD: a novel mucosal adjuvant targeted influenza vaccine. Vaccine. (2008) 26:1243–52. 10.1016/j.vaccine.2007.12.027 18243429

[72] Bernasconi V Bernocchi B Ye L Le MQ Omokanye A Carpentier R. Porous nanoparticles with self-adjuvanting M2e-fusion protein and recombinant hemagglutinin provide strong and broadly protective immunity against influenza virus infections. Front Immunol. (2018) 9:2060. 10.3389/fimmu.2018.02060 30271406 PMC6146233

[73] De Filette M Ramne A Birkett A Lycke N Lowenadler B Min Jou W. The universal influenza vaccine M2e-HBc administered intranasally in combination with the adjuvant CTA1-DD provides complete protection. Vaccine. (2006) 24:544–51. 10.1016/j.vaccine.2005.08.061 16169634

[74] Hajam IA Dar PA Shahnawaz I Jaume JC Lee JH. Bacterial flagellin-a potent immunomodulatory agent. Exp Mol Med. (2017) 49:e373. 10.1038/emm.2017.172 28860663 PMC5628280

[75] Chabot SM Shawi M Eaves-Pyles T Neutra MR. Effects of flagellin on the functions of follicle-associated epithelium. J Infect Dis. (2008) 198:907–10. 10.1086/591056 18721059 PMC5037956

[76] Skountzou I Martin Mdel P Wang B Ye L Koutsonanos D Weldon W. Salmonella flagellins are potent adjuvants for intranasally administered whole inactivated influenza vaccine. Vaccine. (2010) 28:4103–12. 10.1016/j.vaccine.2009.07.058 19654062 PMC3187848

[77] Hong SH Byun YH Nguyen CT Kim SY Seong BL Park S. Intranasal administration of a flagellin-adjuvanted inactivated influenza vaccine enhances mucosal immune responses to protect mice against lethal infection. Vaccine. (2012) 30:466–74. 10.1016/j.vaccine.2011.10.058 22051136

[78] Wang BZ Xu R Quan FS Kang SM Wang L Compans RW. Intranasal immunization with influenza VLPs incorporating membrane-anchored flagellin induces strong heterosubtypic protection. PLoS ONE. (2010) 5:e13972. 10.1371/journal.pone.0013972 21124769 PMC2993933

[79] Wang L Wang YC Feng H Ahmed T Compans RW Wang BZ. Virus-like particles containing the tetrameric ectodomain of influenza matrix protein 2 and flagellin induce heterosubtypic protection in mice. Biomed Res Int. (2013) 2013:686549. 10.1155/2013/686549 23984396 PMC3745920

[80] Wang BZ Gill HS He C Ou C Wang L Wang YC. Microneedle delivery of an M2e-TLR5 ligand fusion protein to skin confers broadly cross-protective influenza immunity. J Control Release. (2014) 178:1–7. 10.1016/j.jconrel.2014.01.002 24417966 PMC3951682

[81] Wang C Zhu W Luo Y Wang BZ. Gold nanoparticles conjugating recombinant influenza hemagglutinin trimers and flagellin enhanced mucosal cellular immunity. Nanomedicine. (2018) 14:1349–60. 10.1016/j.nano.2018.03.007 29649593 PMC6177327

[82] Wang C Zhu W Wang BZ. Dual-linker gold nanoparticles as adjuvanting carriers for multivalent display of recombinant influenza hemagglutinin trimers and flagellin improve the immunological responses *in vivo* and *in vitro*. Int J Nanomedicine. (2017) 12:4747–62. 10.2147/IJN.S137222 28740382 PMC5503497

[83] Chaung HC Cheng LT Hung LH Tsai PC Skountzou I Wang B. Salmonella flagellin enhances mucosal immunity of avian influenza vaccine in chickens. Vet Microbiol. (2012) 157:69–77. 10.1016/j.vetmic.2011.12.014 22226542

[84] Song L Xiong D Song H Wu L Zhang M Kang X Mucosal and systemic immune responses to influenza H7N9 antigen HA1-2 co-delivered intranasally with flagellin or polyethyleneimine in mice and chickens. Front Immunol. (2017) 8:326 10.3389/fimmu.2017.00326 28424686 PMC5380672

[85] Tregoning JS Russell RF Kinnear E. Adjuvanted influenza vaccines. Hum Vaccin Immunother. (2018) 14:550–64. 10.1080/21645515.2017.1415684 29232151 PMC5861793

[86] Turley CB Rupp RE Johnson C Taylor DN Wolfson J Tussey L. Safety and immunogenicity of a recombinant M2e-flagellin influenza vaccine (STF2.4xM2e) in healthy adults. Vaccine. (2011) 29:5145–52. 10.1016/j.vaccine.2011.05.041 21624416

[87] Burt D Mallett C Plante M Zimmermann J Torossian K Fries L. Proteosome-adjuvanted intranasal influenza vaccines: advantages, progress and future considerations. Expert Rev Vaccines. (2011) 10:365–75. 10.1586/erv.10.172 21434804

[88] Plante M Jones T Allard F Torossian K Gauthier J St-Felix N. Nasal immunization with subunit proteosome influenza vaccines induces serum HAI, mucosal IgA and protection against influenza challenge. Vaccine. (2001) 20:218–25. 10.1016/S0264-410X(01)00268-7 11567767

[89] Jones T Cyr S Allard F Bellerose N Lowell GH Burt DS. Protollin: a novel adjuvant for intranasal vaccines. Vaccine. (2004) 22:3691–7. 10.1016/j.vaccine.2004.03.035 15315848

[90] Jones T Allard F Cyr SL Tran SP Plante M Gauthier J. A nasal Proteosome influenza vaccine containing baculovirus-derived hemagglutinin induces protective mucosal and systemic immunity. Vaccine. (2003) 21:3706–12. 10.1016/S0264-410X(03)00387-6 12922101

[91] Lambkin-Williams R Gelder C Broughton R Mallett CP Gilbert AS Mann A. An intranasal proteosome-adjuvanted trivalent influenza vaccine is safe, immunogenic & efficacious in the human viral influenza challenge model. Serum IgG & mucosal IgA are important correlates of protection against illness associated with infection. PLoS ONE. (2016) 11:e0163089. 10.1371/journal.pone.0163089 28005959 PMC5179046

[92] Van Braeckel-Budimir N Haijema BJ Leenhouts K Bacterium-like particles for efficient immune stimulation of existing vaccines and new subunit vaccines in mucosal applications. Front Immunol. (2013) 4:282 10.3389/fimmu.2013.00282 24062748 PMC3775300

[93] Saluja V Amorij JP van Roosmalen ML Leenhouts K Huckriede A Hinrichs WL. Intranasal delivery of influenza subunit vaccine formulated with GEM particles as an adjuvant. AAPS J. (2010) 12:109–16. 10.1208/s12248-009-9168-2 20058113 PMC2844513

[94] de Haan A Haijema BJ Voorn P Meijerhof T van Roosmalen ML Leenhouts K. Bacterium-like particles supplemented with inactivated influenza antigen induce cross-protective influenza-specific antibody responses through intranasal administration. Vaccine. (2012) 30:4884–91. 10.1016/j.vaccine.2012.04.032 22537989

[95] Keijzer C Haijema BJ Meijerhof T Voorn P de Haan A Leenhouts K. Inactivated influenza vaccine adjuvanted with bacterium-like particles induce systemic and mucosal influenza A virus specific T-cell and B-cell responses after nasal administration in a TLR2 dependent fashion. Vaccine. (2014) 32:2904–10. 10.1016/j.vaccine.2014.02.019 24598720

[96] Lee TY Kim CU Bae EH Seo SH Jeong DG Yoon SW. Outer membrane vesicles harboring modified lipid A moiety augment the efficacy of an influenza vaccine exhibiting reduced endotoxicity in a mouse model. Vaccine. (2017) 35:586–95. 10.1016/j.vaccine.2016.12.025 28024958 PMC7115551

[97] Bernasconi V Norling K Bally M Hook F Lycke NY. Mucosal vaccine development based on liposome technology. J Immunol Res. (2016) 2016:5482087. 10.1155/2016/5482087 28127567 PMC5227169

[98] Corthesy B Bioley G. Lipid-Based Particles: versatile delivery systems for mucosal vaccination against infection. Front Immunol. (2018) 9:431. 10.3389/fimmu.2018.00431 29563912 PMC5845866

[99] Joseph A Louria-Hayon I Plis-Finarov A Zeira E Zakay-Rones Z Raz E. Liposomal immunostimulatory DNA sequence (ISS-ODN): an efficient parenteral and mucosal adjuvant for influenza and hepatitis B vaccines. Vaccine. (2002) 20:3342–54. 10.1016/S0264-410X(02)00295-5 12213404

[100] Qu W Li N Yu R Zuo W Fu T Fei W. Cationic DDA/TDB liposome as a mucosal vaccine adjuvant for uptake by dendritic cells *in vitro* induces potent humoural immunity. Artif Cells Nanomed Biotechnol. 46:852–60. 10.1080/21691401.2018.1438450 29447484

[101] Christensen D Foged C Rosenkrands I Lundberg CV Andersen P Agger EM. CAF01 liposomes as a mucosal vaccine adjuvant: *in vitro* and *in vivo* investigations. Int J Pharm. (2010) 390:19–24. 10.1016/j.ijpharm.2009.10.043 19879346

[102] Even-Or O Joseph A Itskovitz-Cooper N Samira S Rochlin E Eliyahu H. A new intranasal influenza vaccine based on a novel polycationic lipid-ceramide carbamoyl-spermine (CCS). II. Studies in mice and ferrets and mechanism of adjuvanticity. Vaccine. (2011) 29:2474–86. 10.1016/j.vaccine.2011.01.009 21251901

[103] Joseph A Itskovitz-Cooper N Samira S Flasterstein O Eliyahu H Simberg D. A new intranasal influenza vaccine based on a novel polycationic lipid-ceramide carbamoyl-spermine (CCS) I. Immunogenicity and efficacy studies in mice. Vaccine. (2006) 24:3990–4006. 10.1016/j.vaccine.2005.12.017 16516356

[104] Even-Or O Samira S Ellis R Kedar E Barenholz Y. Adjuvanted influenza vaccines. Expert Rev Vaccines. (2013) 12:1095–108. 10.1586/14760584.2013.825445 24053401

[105] Wang D Christopher ME Nagata LP Zabielski MA Li H Wong JP. Intranasal immunization with liposome-encapsulated plasmid DNA encoding influenza virus hemagglutinin elicits mucosal, cellular and humoral immune responses. J Clin Virol. (2004) 31(Suppl. 1):S99–106. 10.1016/j.jcv.2004.09.013 15567101

[106] Ninomiya A Ogasawara K Kajino K Takada A Kida H. Intranasal administration of a synthetic peptide vaccine encapsulated in liposome together with an anti-CD40 antibody induces protective immunity against influenza A virus in mice. Vaccine. (2002) 20:3123–9. 10.1016/S0264-410X(02)00261-X 12163263

[107] Adler-Moore J Munoz M Kim H Romero J Tumpey T Zeng H. Characterization of the murine Th2 response to immunization with liposomal M2e influenza vaccine. Vaccine. (2011) 29:4460–8. 10.1016/j.vaccine.2011.04.040 21545821

[108] Scorza FB Pardi N. New kids on the block: RNA-based influenza virus vaccines. Vaccines. (2018) 6:20 10.3390/vaccines6020020 29614788 PMC6027361

[109] Reichmuth AM Oberli MA Jaklenec A Langer R Blankschtein D. mRNA vaccine delivery using lipid nanoparticles. Ther Deliv. (2016) 7:319–34. 10.4155/tde-2016-0006 27075952 PMC5439223

[110] Magini D Giovani C Mangiavacchi S Maccari S Cecchi R Ulmer JB. Self-amplifying mRNA vaccines expressing multiple conserved influenza antigens confer protection against homologous and heterosubtypic viral challenge. PLoS ONE. (2016) 11:e0161193. 10.1371/journal.pone.0161193 27525409 PMC4985159

[111] Bahl K Senn JJ Yuzhakov O Bulychev A Brito LA Hassett KJ. Preclinical and clinical demonstration of immunogenicity by mRNA vaccines against H10N8 and H7N9 influenza viruses. Mol Ther. (2017) 25:1316–27. 10.1016/j.ymthe.2017.03.035 28457665 PMC5475249

[112] Pardi N Parkhouse K Kirkpatrick E McMahon M Zost SJ Mui BL. Nucleoside-modified mRNA immunization elicits influenza virus hemagglutinin stalk-specific antibodies. Nat Commun. (2018) 9:3361. 10.1038/s41467-018-05482-0 30135514 PMC6105651

[113] Phua KK Staats HF Leong KW Nair SK. Intranasal mRNA nanoparticle vaccination induces prophylactic and therapeutic anti-tumor immunity. Sci Rep. (2014) 4:5128. 10.1038/srep05128 24894817 PMC4044635

[114] Ebensen T Debarry J Pedersen GK Blazejewska P Weissmann S Schulze K. Mucosal administration of cycle-di-nucleotide-adjuvanted virosomes efficiently induces protection against influenza H5N1 in mice. Front Immunol. (2017) 8:1223. 10.3389/fimmu.2017.01223 29033942 PMC5624999

[115] Moser C Muller M Kaeser MD Weydemann U Amacker M. Influenza virosomes as vaccine adjuvant and carrier system. Expert Rev Vaccines. (2013) 12:779–91. 10.1586/14760584.2013.811195 23885823

[116] Drane D Gittleson C Boyle J Maraskovsky E. ISCOMATRIX adjuvant for prophylactic and therapeutic vaccines. Expert Rev Vaccines. (2007) 6:761–72. 10.1586/14760584.6.5.761 17931156

[117] Eliasson DG Helgeby A Schon K Nygren C El-Bakkouri K Fiers W. A novel non-toxic combined CTA1-DD and ISCOMS adjuvant vector for effective mucosal immunization against influenza virus. Vaccine. (2011) 29:3951–61. 10.1016/j.vaccine.2011.03.090 21481325

[118] Coulter A Harris R Davis R Drane D Cox J Ryan D. Intranasal vaccination with ISCOMATRIX adjuvanted influenza vaccine. Vaccine. (2003) 21:946–9. 10.1016/S0264-410X(02)00545-5 12547607

[119] Suartha IN Suartini GAA Wirata IW Dewi N Putra GNN Kencana GAY. Intranasal administration of inactivated avian influenza virus of H5N1 subtype vaccine-induced systemic immune response in chicken and mice. Vet World. (2018) 11:221–6. 10.14202/vetworld.2018.221-226 29657407 PMC5891878

[120] Mizuno D Ide-Kurihara M Ichinomiya T Kubo I Kido H. Modified pulmonary surfactant is a potent adjuvant that stimulates the mucosal IgA production in response to the influenza virus antigen. J Immunol. (2006) 176:1122–30. 10.4049/jimmunol.176.2.1122 16394001

[121] Mizuno D Kimoto T Takei T Fukuta A Shinahara W Takahashi E. Surfactant protein C is an essential constituent for mucosal adjuvanticity of Surfacten, acting as an antigen delivery vehicle and inducing both local and systemic immunity. Vaccine. (2011) 29:5368–78. 10.1016/j.vaccine.2011.05.090 21669246

[122] Kimoto T Mizuno D Takei T Kunimi T Ono S Sakai S. Intranasal influenza vaccination using a new synthetic mucosal adjuvant SF-10: induction of potent local and systemic immunity with balanced Th1 and Th2 responses. Influenza Other Respir Viruses. (2013) 7:1218–26. 10.1111/irv.12124 23710832 PMC3933764

[123] Kim H Kimoto T Sakai S Takahashi E Kido H. Adjuvanting influenza hemagglutinin vaccine with a human pulmonary surfactant-mimicking synthetic compound SF-10 induces local and systemic cell-mediated immunity in mice. PLoS ONE. (2018) 13:e0191133. 10.1371/journal.pone.0191133 29370185 PMC5784949

[124] Mizuno D Kimoto T Sakai S Takahashi E Kim H Kido H. Induction of systemic and mucosal immunity and maintenance of its memory against influenza A virus by nasal vaccination using a new mucosal adjuvant SF-10 derived from pulmonary surfactant in young cynomolgus monkeys. Vaccine. (2016) 34:1881–8. 10.1016/j.vaccine.2016.02.061 26954466

[125] Maltais AK Stittelaar KJ Veldhuis Kroeze EJ van Amerongen G Dijkshoorn ML Krestin GP. Intranasally administered Endocine formulated 2009 pandemic influenza H1N1 vaccine induces broad specific antibody responses and confers protection in ferrets. Vaccine. (2014) 32:3307–15. 10.1016/j.vaccine.2014.03.061 24690149

[126] Falkeborn T Brave A Larsson M Akerlind B Schroder U Hinkula J Endocine, N3OA and N3OASq; three mucosal adjuvants that enhance the immune response to nasal influenza vaccination. PLoS ONE. (2013) 8:e70527 10.1371/journal.pone.0070527 23950951 PMC3738562

[127] Falkeborn T Hinkula J Olliver M Lindberg A Maltais AK The intranasal adjuvant Endocine enhances both systemic and mucosal immune responses in aged mice immunized with influenza antigen. Virol J. (2017) 14:44 10.1186/s12985-017-0698-4 28253901 PMC5335733

[128] Makidon PE Belyakov IM Blanco LP Janczak KW Landers J Bielinska AU. Nanoemulsion mucosal adjuvant uniquely activates cytokine production by nasal ciliated epithelium and induces dendritic cell trafficking. Eur J Immunol. (2012) 42:2073–86. 10.1002/eji.201142346 22653620 PMC3929939

[129] Myc A Kukowska-Latallo JF Smith DM Passmore C Pham T Wong P Nanoemulsion nasal adjuvant W_80_5EC induces dendritic cell engulfment of antigen-primed epithelial cells. Vaccine. (2013) 31:1072–9. 10.1016/j.vaccine.2012.12.033 23273511 PMC3556236

[130] Bielinska AU Gerber M Blanco LP Makidon PE Janczak KW Beer M. Induction of Th17 cellular immunity with a novel nanoemulsion adjuvant. Crit Rev Immunol. (2010) 30:189–99. 10.1615/CritRevImmunol.v30.i2.60 20370629 PMC2860868

[131] Bielinska AU Makidon PE Janczak KW Blanco LP Swanson B Smith DM. Distinct pathways of humoral and cellular immunity induced with the mucosal administration of a nanoemulsion adjuvant. J Immunol. (2014) 192:2722–33. 10.4049/jimmunol.1301424 24532579 PMC3948110

[132] Das SC Hatta M Wilker PR Myc A Hamouda T Neumann G. Nanoemulsion W_80_5EC improves immune responses upon intranasal delivery of an inactivated pandemic H1N1 influenza vaccine. Vaccine. (2012) 30:6871–7. 10.1016/j.vaccine.2012.09.007 22989689 PMC3488283

[133] Hamouda T Sutcliffe JA Ciotti S Baker JR Jr. Intranasal immunization of ferrets with commercial trivalent influenza vaccines formulated in a nanoemulsion-based adjuvant. Clin Vaccine Immunol. (2011) 18:1167–75. 10.1128/CVI.00035-11 21543588 PMC3147313

[134] Stanberry LR Simon JK Johnson C Robinson PL Morry J Flack MR. Safety and immunogenicity of a novel nanoemulsion mucosal adjuvant W_80_5EC combined with approved seasonal influenza antigens. Vaccine. (2012) 30:307–16. 10.1016/j.vaccine.2011.10.094 22079079

[135] Brazzoli M Magini D Bonci A Buccato S Giovani C Kratzer R. Induction of broad-based immunity and protective efficacy by self-amplifying mRNA vaccines encoding influenza virus hemagglutinin. J Virol. (2016) 90:332–44. 10.1128/JVI.01786-15 26468547 PMC4702536

[136] Quan FS Lee YT Kim KH Kim MC Kang SM. Progress in developing virus-like particle influenza vaccines. Expert Rev Vaccines. (2016) 15:1281–93. 10.1080/14760584.2016.1175942 27058302 PMC5093318

[137] Kang SM Song JM Quan FS Compans RW. Influenza vaccines based on virus-like particles. Virus Res. (2009) 143:140–6. 10.1016/j.virusres.2009.04.005 19374929 PMC2753524

[138] Xia M Tan M Wei C Zhong W Wang L McNeal M. A candidate dual vaccine against influenza and noroviruses. Vaccine. (2011) 29:7670–7. 10.1016/j.vaccine.2011.07.139 21839795 PMC3190067

[139] Perrone LA Ahmad A Veguilla V Lu X Smith G Katz JM. Intranasal vaccination with 1918 influenza virus-like particles protects mice and ferrets from lethal 1918 and H5N1 influenza virus challenge. J Virol. (2009) 83:5726–34. 10.1128/JVI.00207-09 19321609 PMC2681940

[140] Quan FS Huang C Compans RW Kang SM. Virus-like particle vaccine induces protective immunity against homologous and heterologous strains of influenza virus. J Virol. (2007) 81:3514–24. 10.1128/JVI.02052-06 17251294 PMC1866067

[141] Song JM Wang BZ Park KM Van Rooijen N Quan FS Kim MC. Influenza virus-like particles containing M2 induce broadly cross protective immunity. PLoS ONE. (2011) 6:e14538. 10.1371/journal.pone.0014538 21267073 PMC3022578

[142] Schwartzman LM Cathcart AL Pujanauski LM Qi L Kash JC Taubenberger JK. An intranasal virus-like particle vaccine broadly protects mice from multiple subtypes of influenza A virus. MBio. (2015) 6:e01044. 10.1128/mBio.01044-15 26199334 PMC4513078

[143] Chen S Zheng D Li C Zhang W Xu W Liu X. Protection against multiple subtypes of influenza viruses by virus-like particle vaccines based on a hemagglutinin conserved epitope. Biomed Res Int. (2015) 2015:901817. 10.1155/2015/901817 25767809 PMC4341857

[144] Zheng D Chen S Qu D Chen J Wang F Zhang R. Influenza H7N9 LAH-HBc virus-like particle vaccine with adjuvant protects mice against homologous and heterologous influenza viruses. Vaccine. (2016) 34:6464–71. 10.1016/j.vaccine.2016.11.026 27866773

[145] Bessa J Schmitz N Hinton HJ Schwarz K Jegerlehner A Bachmann MF. Efficient induction of mucosal and systemic immune responses by virus-like particles administered intranasally: implications for vaccine design. Eur J Immunol. (2008) 38:114–26. 10.1002/eji.200636959 18081037

[146] Elaish M Kang KI Xia M Ali A Shany SA Wang L. Immunogenicity and protective efficacy of the norovirus P particle-M2e chimeric vaccine in chickens. Vaccine. (2015) 33:4901–9. 10.1016/j.vaccine.2015.07.049 26232342

[147] Herve PL Raliou M Bourdieu C Dubuquoy C Petit-Camurdan A Bertho N. A novel subnucleocapsid nanoplatform for mucosal vaccination against influenza virus that targets the ectodomain of matrix protein 2. J Virol. (2014) 88:325–38. 10.1128/JVI.01141-13 24155388 PMC3911713

[148] Bessa J Jegerlehner A Hinton HJ Pumpens P Saudan P Schneider P. Alveolar macrophages and lung dendritic cells sense RNA and drive mucosal IgA responses. J Immunol. (2009) 183:3788–99. 10.4049/jimmunol.0804004 19710454

[149] Bessa J Zabel F Link A Jegerlehner A Hinton HJ Schmitz N. Low-affinity B cells transport viral particles from the lung to the spleen to initiate antibody responses. Proc Natl Acad Sci USA. (2012) 109:20566–71. 10.1073/pnas.1206970109 23169669 PMC3528531

[150] Schneider-Ohrum K Giles BM Weirback HK Williams BL DeAlmeida DR Ross TM. Adjuvants that stimulate TLR3 or NLPR3 pathways enhance the efficiency of influenza virus-like particle vaccines in aged mice. Vaccine. (2011) 29:9081–92. 10.1016/j.vaccine.2011.09.051 21963872 PMC6690196

[151] Mohan T Deng L Wang BZ. CCL28 chemokine: an anchoring point bridging innate and adaptive immunity. Int Immunopharmacol. (2017) 51:165–70. 10.1016/j.intimp.2017.08.012 28843907 PMC5755716

[152] Xia Y Fan Q Hao D Wu J Ma G Su Z. Chitosan-based mucosal adjuvants: sunrise on the ocean. Vaccine. (2015) 33:5997–6010. 10.1016/j.vaccine.2015.07.101 26271831 PMC7185844

[153] Carroll EC Jin L Mori A Munoz-Wolf N Oleszycka E Moran HBT. The vaccine adjuvant chitosan promotes cellular immunity via DNA sensor cGAS-STING-dependent induction of type I interferons. Immunity. (2016) 44:597–608. 10.1016/j.immuni.2016.02.004 26944200 PMC4852885

[154] Liu Q Zheng X Zhang C Shao X Zhang X Zhang Q. Conjugating influenza A. (H1N1) antigen to N-trimethylaminoethylmethacrylate chitosan nanoparticles improves the immunogenicity of the antigen after nasal administration. J Med Virol. (2015) 87:1807–15. 10.1002/jmv.24253 25959372

[155] Amidi M Romeijn SG Verhoef JC Junginger HE Bungener L Huckriede A. N-trimethyl chitosan (TMC) nanoparticles loaded with influenza subunit antigen for intranasal vaccination: biological properties and immunogenicity in a mouse model. Vaccine. (2007) 25:144–53. 10.1016/j.vaccine.2006.06.086 16973248

[156] Wu Y Wei W Zhou M Wang Y Wu J Ma G. Thermal-sensitive hydrogel as adjuvant-free vaccine delivery system for H5N1 intranasal immunization. Biomaterials. (2012) 33:2351–60. 10.1016/j.biomaterials.2011.11.068 22192540

[157] Mann AJ Noulin N Catchpole A Stittelaar KJ de Waal L Veldhuis Kroeze EJ. Intranasal H5N1 vaccines, adjuvanted with chitosan derivatives, protect ferrets against highly pathogenic influenza intranasal and intratracheal challenge. PLoS ONE. (2014) 9:e93761. 10.1371/journal.pone.0093761 24850536 PMC4029577

[158] Sawaengsak C Mori Y Yamanishi K Srimanote P Chaicumpa W Mitrevej A. Intranasal chitosan-DNA vaccines that protect across influenza virus subtypes. Int J Pharm. (2014) 473:113–25. 10.1016/j.ijpharm.2014.07.005 24998507

[159] Sui Z Chen Q Wu R Zhang H Zheng M Wang H. Cross-protection against influenza virus infection by intranasal administration of M2-based vaccine with chitosan as an adjuvant. Arch Virol. (2010) 155:535–44. 10.1007/s00705-010-0621-4 20195654

[160] Moon HJ Lee JS Talactac MR Chowdhury MY Kim JH Park ME. Mucosal immunization with recombinant influenza hemagglutinin protein and poly gamma-glutamate/chitosan nanoparticles induces protection against highly pathogenic influenza A virus. Vet Microbiol. (2012) 160:277–89. 10.1016/j.vetmic.2012.05.035 22763171

[161] Gupta NK Tomar P Sharma V Dixit VK Development and characterization of chitosan coated poly-(ε-caprolactone) nanoparticulate system for effective immunization against influenza. Vaccine. (2011) 29:9026–37. 10.1016/j.vaccine.2011.09.033 21939718

[162] Alkie TN Yitbarek A Taha-Abdelaziz K Astill J Sharif S. Characterization of immunogenicity of avian influenza antigens encapsulated in PLGA nanoparticles following mucosal and subcutaneous delivery in chickens. PLoS ONE. (2018) 13:e0206324. 10.1371/journal.pone.0206324 30383798 PMC6211703

[163] McCullough KC Bassi I Milona P Suter R Thomann-Harwood L Englezou P. Self-replicating replicon-RNA delivery to dendritic cells by chitosan-nanoparticles for translation *in vitro* and *in vivo*. Mol Ther Nucleic Acids. (2014) 3:e173. 10.1038/mtna.2014.24 25004099 PMC4121514

[164] Read RC Naylor SC Potter CW Bond J Jabbal-Gill I Fisher A. Effective nasal influenza vaccine delivery using chitosan. Vaccine. (2005) 23:4367–74. 10.1016/j.vaccine.2005.04.021 15916838

[165] Smith A Perelman M Hinchcliffe M. Chitosan: a promising safe and immune-enhancing adjuvant for intranasal vaccines. Hum Vaccin Immunother. (2014) 10:797–807. 10.4161/hv.27449 24346613 PMC4130252

[166] Jia Y Krishnan L Omri A. Nasal and pulmonary vaccine delivery using particulate carriers. Expert Opin Drug Deliv. (2015) 12:993–1008. 10.1517/17425247.2015.1044435 25952104

[167] Noh HJ Chowdhury MY Cho S Kim JH Park HS Kim CJ. Programming of influenza vaccine broadness and persistence by mucoadhesive polymer-based adjuvant systems. J Immunol. (2015) 195:2472–82. 10.4049/jimmunol.1500492 26216889

[168] Okamoto S Matsuura M Akagi T Akashi M Tanimoto T Ishikawa T. Poly(γ-glutamic acid) nano-particles combined with mucosal influenza virus hemagglutinin vaccine protects against influenza virus infection in mice. Vaccine. (2009) 27:5896–905. 10.1016/j.vaccine.2009.07.037 19647814

[169] Torrieri-Dramard L Lambrecht B Ferreira HL Van den Berg T Klatzmann D Bellier B. Intranasal DNA vaccination induces potent mucosal and systemic immune responses and cross-protective immunity against influenza viruses. Mol Ther. (2011) 19:602–11. 10.1038/mt.2010.222 20959813 PMC3048174

[170] Li M Zhao M Fu Y Li Y Gong T Zhang Z. Enhanced intranasal delivery of mRNA vaccine by overcoming the nasal epithelial barrier via intra- and paracellular pathways. J Control Release. (2016) 228:9-19. 10.1016/j.jconrel.2016.02.043 26941035

[171] Demoulins T Milona P Englezou PC Ebensen T Schulze K Suter R. Polyethylenimine-based polyplex delivery of self-replicating RNA vaccines. Nanomedicine. (2016) 12:711–22. 10.1016/j.nano.2015.11.001 26592962

[172] Vogel AB Lambert L Kinnear E Busse D Erbar S Reuter KC. Self-Amplifying RNA vaccines give equivalent protection against influenza to mRNA vaccines but at much lower doses. Mol Ther. (2018) 26:446–55. 10.1016/j.ymthe.2017.11.017 29275847 PMC5835025

[173] Qin T Yin Y Huang L Yu Q Yang Q. H9N2 influenza whole inactivated virus combined with polyethyleneimine strongly enhances mucosal and systemic immunity after intranasal immunization in mice. Clin Vaccine Immunol. (2015) 22:421–9. 10.1128/CVI.00778-14 25673304 PMC4375346

[174] Mann JF McKay PF Arokiasamy S Patel RK Klein K Shattock RJ. Pulmonary delivery of DNA vaccine constructs using deacylated PEI elicits immune responses and protects against viral challenge infection. J Control Release. (2013) 170:452–9. 10.1016/j.jconrel.2013.06.004 23774102 PMC3767111

[175] Csaba N Garcia-Fuentes M Alonso MJ. Nanoparticles for nasal vaccination. Adv Drug Deliv Rev. (2009) 61:140–57. 10.1016/j.addr.2008.09.005 19121350

[176] Singh SM Alkie TN Abdelaziz KT Hodgins DC Novy A Nagy E. Characterization of immune responses to an inactivated avian influenza virus vaccine adjuvanted with nanoparticles containing CpG ODN. Viral Immunol. (2016) 29:269–75. 10.1089/vim.2015.0144 27077969

[177] Sakuma S Suita M Yamamoto T Masaoka Y Kataoka M Yamashita S. Performance of cell-penetrating peptide-linked polymers physically mixed with poorly membrane-permeable molecules on cell membranes. Eur J Pharm Biopharm. (2012) 81:64–73. 10.1016/j.ejpb.2012.01.008 22306700

[178] Sakuma S Suita M Inoue S Marui Y Nishida K Masaoka Y. Cell-penetrating peptide-linked polymers as carriers for mucosal vaccine delivery. Mol Pharm. (2012) 9:2933–41. 10.1021/mp300329r 22953762

[179] Miyata K Mohri K Egawa T Endo R Morimoto N Ochiai K. Demonstration of D-octaarginine-linked polymers as promising adjuvants for mucosal vaccination through influenza virus challenge. Bioconjug Chem. (2016) 27:1865–71. 10.1021/acs.bioconjchem.6b00283 27463562

[180] Salazar-Gonzalez JA Gonzalez-Ortega O Rosales-Mendoza S. Gold nanoparticles and vaccine development. Expert Rev Vaccines. (2015) 14:1197–211. 10.1586/14760584.2015.1064772 26152550

[181] Tao W Ziemer KS Gill HS. Gold nanoparticle-M2e conjugate coformulated with CpG induces protective immunity against influenza A virus. Nanomedicine. (2014) 9:237–51. 10.2217/nnm.13.58 23829488 PMC3958969

[182] Tao W Hurst BL Shakya AK Uddin MJ Ingrole RS Hernandez-Sanabria M. Consensus M2e peptide conjugated to gold nanoparticles confers protection against H1N1, H3N2 and H5N1 influenza A viruses. Antiviral Res. (2017) 141:62–72. 10.1016/j.antiviral.2017.01.021 28161578 PMC5572660

[183] Takaki H Kure S Oshiumi H Sakoda Y Suzuki T Ainai A. Toll-like receptor 3 in nasal CD103^+^ dendritic cells is involved in immunoglobulin A production. Mucosal Immunol. (2018) 11:82–96. 10.1038/mi.2017.48 28612840

[184] Ichinohe T Watanabe I Ito S Fujii H Moriyama M Tamura S. Synthetic double-stranded RNA poly(I:C) combined with mucosal vaccine protects against influenza virus infection. J Virol. (2005) 79:2910–9. 10.1128/JVI.79.5.2910-2919.2005 15709010 PMC548446

[185] Goff PH Eggink D Seibert CW Hai R Martinez-Gil L Krammer F. Adjuvants and immunization strategies to induce influenza virus hemagglutinin stalk antibodies. PLoS ONE. (2013) 8:e79194. 10.1371/journal.pone.0079194 24223176 PMC3819267

[186] Liang J Fu J Kang H Lin J Yu Q Yang Q. Comparison of 3 kinds of Toll-like receptor ligands for inactivated avian H5N1 influenza virus intranasal immunization in chicken. Poult Sci. (2013) 92:2651–60. 10.3382/ps.2013-03193 24046412

[187] Ichinohe T Tamura S Kawaguchi A Ninomiya A Imai M Itamura S. Cross-protection against H5N1 influenza virus infection is afforded by intranasal inoculation with seasonal trivalent inactivated influenza vaccine. J Infect Dis. (2007) 196:1313–20. 10.1086/521304 17922395 PMC7110255

[188] Overton ET Goepfert PA Cunningham P Carter WA Horvath J Young D. Intranasal seasonal influenza vaccine and a TLR-3 agonist, rintatolimod, induced cross-reactive IgA antibody formation against avian H5N1 and H7N9 influenza HA in humans. Vaccine. (2014) 32:5490–5. 10.1016/j.vaccine.2014.07.078 25128802

[189] Takaki H Takashima K Oshiumi H Ainai A Suzuki T Hasegawa H. cGAMP promotes germinal center formation and production of IgA in nasal-associated lymphoid tissue. Med Sci. (2017) 5:35 10.3390/medsci5040035 29258267 PMC5753664

[190] Madhun AS Haaheim LR Nostbakken JK Ebensen T Chichester J Yusibov V. Intranasal c-di-GMP-adjuvanted plant-derived H5 influenza vaccine induces multifunctional Th1 CD4^+^ cells and strong mucosal and systemic antibody responses in mice. Vaccine. (2011) 29:4973–82. 10.1016/j.vaccine.2011.04.094 21600260

[191] Svindland SC Pedersen GK Pathirana RD Bredholt G Nostbakken JK Jul-Larsen A. A study of chitosan and c-di-GMP as mucosal adjuvants for intranasal influenza H5N1 vaccine. Influenza Other Respir Viruses. (2013) 7:1181–93. 10.1111/irv.12056 23170900 PMC4634239

[192] Sanchez MV Ebensen T Schulze K Cargnelutti D Blazejewska P Scodeller EA. Intranasal delivery of influenza rNP adjuvanted with c-di-AMP induces strong humoral and cellular immune responses and provides protection against virus challenge. PLoS ONE. (2014) 9:e104824. 10.1371/journal.pone.0104824 25140692 PMC4139298

[193] Klinman DM. Immunotherapeutic uses of CpG oligodeoxynucleotides. Nat Rev Immunol. (2004) 4:249–58. 10.1038/nri1329 15057783

[194] Moldoveanu Z Love-Homan L Huang WQ Krieg AM. CpG DNA, a novel immune enhancer for systemic and mucosal immunization with influenza virus. Vaccine. (1998) 16:1216–24. 10.1016/S0264-410X(98)80122-9 9682382

[195] Fu J Liang J Kang H Lin J Yu Q Yang Q. Effects of different CpG oligodeoxynucleotides with inactivated avian H5N1 influenza virus on mucosal immunity of chickens. Poult Sci. (2013) 92:2866–75. 10.3382/ps.2013-03205 24135589

[196] Zacharias ZR Ross KA Hornick EE Goodman JT Narasimhan B Waldschmidt TJ. Polyanhydride nanovaccine induces robust pulmonary B and T cell immunity and confers protection against homologous and heterologous influenza A virus infections. Front Immunol. (2018) 9:1953. 10.3389/fimmu.2018.01953 30233573 PMC6127617

[197] Qin T Yin Y Yu Q Huang L Wang X Lin J. CpG oligodeoxynucleotides facilitate delivery of whole inactivated H9N2 influenza virus via transepithelial dendrites of dendritic cells in nasal mucosa. J Virol. (2015) 89:5904–18. 10.1128/JVI.00296-15 25810544 PMC4442437

[198] Wang X Meng D. Innate endogenous adjuvants prime to desirable immune responses via mucosal routes. Protein Cell. (2015) 6:170–84. 10.1007/s13238-014-0125-1 25503634 PMC4348248

[199] Arulanandam BP O'Toole M Metzger DW. Intranasal interleukin-12 is a powerful adjuvant for protective mucosal immunity. J Infect Dis. (1999) 180:940–9. 10.1086/314996 10479116

[200] Bracci L Canini I Puzelli S Sestili P Venditti M Spada M. Type I IFN is a powerful mucosal adjuvant for a selective intranasal vaccination against influenza virus in mice and affects antigen capture at mucosal level. Vaccine. (2005) 23:2994–3004. 10.1016/j.vaccine.2004.12.006 15811645

[201] Kayamuro H Yoshioka Y Abe Y Arita S Katayama K Nomura T. Interleukin-1 family cytokines as mucosal vaccine adjuvants for induction of protective immunity against influenza virus. J Virol. (2010) 84:12703–12. 10.1128/JVI.01182-10 20881038 PMC3004317

[202] Khan T Heffron CL High KP Roberts PC. Membrane-bound IL-12 and IL-23 serve as potent mucosal adjuvants when co-presented on whole inactivated influenza vaccines. Virol J. (2014) 11:78. 10.1186/1743-422X-11-78 24884849 PMC4036309

[203] Mohan T Kim J Berman Z Wang S Compans RW Wang BZ. Co-delivery of GPI-anchored CCL28 and influenza HA in chimeric virus-like particles induces cross-protective immunity against H3N2 viruses. J Control Release. (2016) 233:208–19. 10.1016/j.jconrel.2016.05.021 27178810 PMC4912934

[204] Liu J Ren Z Wang H Zhao Y Wilker PR Yu Z. Influenza virus-like particles composed of conserved influenza proteins and GPI-anchored CCL28/GM-CSF fusion proteins enhance protective immunity against homologous and heterologous viruses. Int Immunopharmacol. (2018) 63:119–28. 10.1016/j.intimp.2018.07.011 30081250

[205] Couch RB Atmar RL Cate TR Quarles JM Keitel WA Arden NH. Contrasting effects of type I interferon as a mucosal adjuvant for influenza vaccine in mice and humans. Vaccine. (2009) 27:5344–8. 10.1016/j.vaccine.2009.06.084 19607949 PMC2778204

[206] Sanchez-Ramon S Conejero L Netea MG Sancho D Palomares O Subiza JL. Trained immunity-based vaccines: a new paradigm for the development of broad-spectrum anti-infectious formulations. Front Immunol. (2018) 9:2936. 10.3389/fimmu.2018.02936 30619296 PMC6304371

[207] Norton EB Clements JD Voss TG Cardenas-Freytag L. Prophylactic administration of bacterially derived immunomodulators improves the outcome of influenza virus infection in a murine model. J Virol. (2010) 84:2983–95. 10.1128/JVI.01805-09 20053748 PMC2826051

[208] Beilharz MW Cummins JM Bennett AL. Protection from lethal influenza virus challenge by oral type 1 interferon. Biochem Biophys Res Commun. (2007) 355:740–4. 10.1016/j.bbrc.2007.02.019 17316562

[209] Lau YF Tang LH Ooi EE. A TLR3 ligand that exhibits potent inhibition of influenza virus replication and has strong adjuvant activity has the potential for dual applications in an influenza pandemic. Vaccine. (2009) 27:1354–64. 10.1016/j.vaccine.2008.12.048 19150474 PMC7115584

[210] Zheng M Qu D Wang H Sun Z Liu X Chen J. Intranasal administration of chitosan against influenza A (H7N9) virus infection in a mouse model. Sci Rep. (2016) 6:28729. 10.1038/srep28729 27353250 PMC4926116

