# Supplementary material for: Innovative Mucosal Vaccine Formulations Against Influenza A Virus Infections
Source: Front Immunol. 2019 Jul 17;10:1605. doi: 10.3389/fimmu.2019.01605 (PMC6650573; doi:10.3389/fimmu.2019.01605)
Supplement: Supplemental Table 1 — Adjuvants/delivery systems tested in intranasal vaccination against IAV infections in pre-clinical and clinical studies. [file Table_1.docx]

**Supplemental Table 1.** Adjuvants/delivery systems tested in intranasal vaccination against IAV infections in pre-clinical and clinical studies

| **Class of adjuvants**  **/**  **Delivery systems** | **Composition** | **Target**  **/**  **Mechanisms of action** | **Pre-clinical tests** | | | | | **Clinical tests (ref)** | **Advantages**  **/**  **Potential limitations** |
| --- | --- | --- | --- | --- | --- | --- | --- | --- | --- |
|  |  |  | **IAV Ags/**  **Vaccine formulations** | **Immunoenhancement^a^** | **Protection^b^** | **Animal model** | **Ref** |  |  |
| **bacterial toxins and derivatives** | **CT** | GM1 | H1N1 WIV | serum/mucosal Abs | Heterologous  (H3N2) | mouse | (1) | licensed  virosome vaccine with LT  withdrawn from the market because of safety issues (Bell’s palsy)  (2) | high adjuvanticity of holotoxins (gold standards for adjuvant potency evaluation)  /  safety issues of holotoxins (development of efficient and safe non-toxic derivatives in animal models and non-human primates) |
|  | **LT(R192G)** |  | H3N2 WIV | serum/mucosal Abs | Heterologous  (H1N1)  [mucosal viral loads]  Heterologous  (H5N1) | mouse | (3) |  |  |
|  | **CTA1** | ? | fusion protein  M2-HA2-CTA1  chitosan-coated γ-PGA nanoparticles  M2 : H1N1/H5N1/H9N2 consensus sequence  HA2: portion of H5N1 HA2 | serum/mucosal Abs  systemic type 1/type 2 cytokine-producing cells | Homologous, Heterologous  (H1N1, H5N1, H5N2, H7N3, H9N2) | mouse | (4) |  |  |
|  | **CTA1-DD** | B cell Ig receptor,  complement receptor | (M2e) VLPs  M2e : 3 repetitions of “human”  consensus sequence | serum Abs | Heterologous  (H3N2) | mouse | (5) |  |  |
|  |  |  | H1N1 HA/NA  ISCOMs | serum/mucosal Abs  systemic type 1/type 2 cytokine-producing cells | ND | mouse | (6) |  |  |
| **PRR ligands**  **PRR LIGANDS** | **Poly (I:C)/ Poly (I:C_12_U)** | TLR3 | H5N1 inactivated virus | serum/mucosal Abs | ND | chicken | (7) | phase I/II  LAIV trivalent vaccine with poly (I:C_12_U):  safe, well tolerated, immunogenic (mucosal humoral responses)  no evaluation of adjuvant effect  (8) | safe in humans (poly (I:C_12_U)), efficient in pre-clinical tests (enhancement of humoral and cellular immunity) |
|  |  |  | H1N1 split virus | serum/mucosal Abs  systemic type 1 cytokine-producing cells | Heterovariant (H1N1) | mouse | (9) |  |  |
|  |  |  | chimeric HAs ± H1 HA  chimeric HAs : HA1 from various subtypes associated with the same H1 HA2 | serum/mucosal Abs | Homologous  (H1N1)  Heterovariant (H1N1) | mouse | (10) |  |  |
|  |  |  | H1N1/H3N2/IBV  split viruses | serum/mucosal Abs  systemic type 1 cytokine-producing cells | Heterologous  (H5N1)  [mucosal viral loads] | mouse | (11) |  |  |
|  | **Flagellin** | TLR5 | H1N1 WIV | serum Abs  systemic type 2 cytokine-producing cells | Homologous (H1N1) | mouse | (12) | N/A  N/A | safe and efficient in pre-clinical tests (enhancement of humoral and cellular immunity) |
|  |  |  | H1N1/H3N2/IBV  split viruses | serum/mucosal Abs  systemic/local type 2 cytokine-producing cells | Homologous (H1N1) | mouse | (13) |  |  |
|  |  |  | fusion protein  flagellin-M2e  M2e : 4 repetitions of “human”  consensus sequence | serum/mucosal Abs | Homologous,  Heterologous (H1N1, H3N2) | mouse | (14) |  |  |
|  |  |  | H1N1 (HA/M1) VLPs | serum/mucosal Abs  systemic type 1/type 2 cytokine-producing cells | Heterologous  (H3N2) | mouse | (15) |  |  |
|  |  |  | fusion protein  H7N9 HA1-flagellin | serum/mucosal Abs | Homologous (H7N9)  [mucosal viral loads] | chicken | (16) |  |  |
|  | **CpG-ODN** | TLR9 | H9N2 WIV | serum/mucosal Abs | ND | mouse | (17) |  |  |
|  |  | TLR21 | H5N1 inactivated virus | serum/mucosal Abs | Heterovariant (H5N1) | chicken | (7, 18) |  |  |
|  | **c-di-AMP** | STING | H5N1 virosomes | serum/mucosal Abs  systemic type 1/type 2/type 17 cytokine-producing cells | Homologous (H5N1) | mouse | (19) |  |  |
|  |  |  | H1N1 NP | serum/mucosal Abs  systemic type 1/type 2/type 17 cytokine-producing cells | Homologous (H1N1)  [weight loss] | mouse | (20) |  |  |
|  | **cGAMP** |  | H1N1 split virus | serum/mucosal Abs  systemic type 1/type 2/type 17 cytokine-producing cells | ND | mouse | (21) |  |  |
|  | **c-di-GMP** | STING | H5N1 HA | serum/mucosal Abs  systemic type 1/type 2/type 17 cytokine-producing cells | ND | mouse | (22) |  |  |
|  | **Proteosomes/**  **Protollin** | TLR2, TLR4, other PRRs ? | H1N1 split virus | serum/mucosal Abs  systemic type 1 cytokine-producing cells | Homologous (H1N1) | mouse | (23) | phase II  trivalent split vaccine with proteosomes:  safe, well tolerated, immunogenic (systemic and mucosal humoral responses) and protective  no evaluation of adjuvant effect  (24) | safe (proteosomes) in humans and efficient in pre-clinical tests (enhancement of humoral and cellular immunity) |
|  |  |  | H1N1 HA | serum/mucosal Abs  systemic type 1 cytokine-producing cells | Heterovariant (H1N1) | mouse | (25, 26) |  |  |
|  | **Bacterium-like Particles** | TLR2, other PRRs ? | H1N1 or H3N2 split viruses | serum/mucosal Abs  systemic type 1 cytokine-producing cells | Homologous (H1N1) | mouse | (27, 28) | phase I  trivalent split vaccine with bacterium-like particles:  safe, well tolerated, immunogenic,  serum and mucosal humoral responses and systemic cellular responses boosted by the adjuvant  (29) | safe and efficient in humans |
|  | **Outer Membrane Vesicles** | Various PRRs | H1N1/H3N2/IBV split viruses | serum/mucosal Abs  systemic/local type 1 cytokine-producing cells | Homologous  (H1N1)  Heterovariant  (H1N1)  Heterologous  (H5N2) | mouse | (30) | N/A | efficient in pre-clinical tests (enhancement of humoral and cellular immunity), possibility to generate safer outer membrane vesicles with attenuated endotoxicity |
| **lipid-based particles**  **LIPID-BASED PARTICLES** | **Liposomes** | Delivery vehicles | H1N1 split virus  plus CpG-ODN | serum/mucosal Abs  systemic type 1 cytokine-producing cells | Heterovariant (H1N1) [mucosal viral loads] | mouse | (31) | phase II  trivalent split vaccine with liposomes  (32, 33)  phase I  virosome vaccine  (34, 35) | easily customisable to modulate the immunogenic properties, immunogenicity of cationic liposomes  /  relatively poor intrinsic stability, poor intrinsic immunogenicity (anionic/neutral liposomes), potential toxicity of cationic lipids |
|  |  |  | H3N2 or H1N1/H3N2/IBV split viruses  plus α,α’-trehalose 6,6’-dibehenate | serum/mucosal Abs  systemic type 1 cytokine-producing cells | ND | mouse | (36, 37) |  |  |
|  |  |  | H1N1 or H1N1/H3N2/IBV split viruses | serum/mucosal Abs  systemic type 1 cytokine-producing cells | Homologous, Heterovariant (H1N1, H3N2)  [mucosal viral loads] | mouse | (38) |  |  |
|  |  |  | H1N1 HA pDNA | serum/mucosal Abs | Homologous (H1N1) | mouse | (39) |  |  |
|  | **ISCOMs** | Delivery vehicles | H1N1 HA/NA | serum/mucosal Abs  systemic type 1/type 2 cytokine-producing cells | ND | mouse | (6, 40) | yes  (41) | efficient in pre-clinical tests (enhancement of humoral and cellular immunity),  development of formulations incorporating less toxic fractions of saponins |
|  | **Pulmonary Surfactant Derivatives (SF-10)** | Delivery vehicles with mucoadhesive properties | H1N1 split virus | serum/mucosal Abs  local type 1/type 2 cytokine-producing cells  systemic CTLs | Homologous (H1N1) [mucosal viral loads] Heterovariant (H1N1) | mouse | (42, 43) | N/A | safe and efficient in animal models and non-human primates (enhancement of humoral and cellular immunity) |
|  |  |  | H1N1 split virus | serum/mucosal Abs | ND | non-human primates | (44) |  |  |
|  | **Endocine** | Delivery vehicles | H1N1/H3N2/IBV  split viruses | serum/mucosal Abs  systemic type 1 cytokine-producing cells | ND | mouse | (45) | N/A | safe in humans, efficient in pre-clinical tests (enhancement of humoral and cellular immunity) |
|  | **W_80_5EC**  **W_80_5EC** | Delivery vehicles,  PRRs  Delivery vehicles,  PRRs | H1N1 WIV | serum/mucosal Abs | Homologous (H1N1) [weight loss and mucosal viral loads] | mouse | (46) | phase I  trivalent split vaccine with W_80_EC:  safe, well tolerated, immunogenic, serum and mucosal humoral responses boosted by the adjuvant  (47) | safe and efficient in humans |
|  |  |  | H1N1/H3N2/IBV  split viruses | serum Abs | ND | ferret | (48) |  |  |
| **virus-LIKE particles** | **VLPs** | Delivery vehicles, PRRs | (M2e) VLPs  M2e : “human”  consensus sequence | serum Abs | Heterologous (H1N1) | mouse | (49) | N/A | mimic structural nature of virus, highly immunogenic  /  potential poor production efficiency, poor quality consistency and toxicity |
|  |  |  | (M2e) VLPs  M2e : tetrameric “human”  consensus sequence | serum/mucosal Abs  local/systemic type 1/type 2 cytokine-producing cells | Homologous (H3N2) | mouse | (50) |  |  |
|  |  |  | H1N1 (M2e) VLPs  1 or 3 repetitions of a H1N1 sequence | serum/mucosal Abs | Homologous (H1N1) | mouse | (51) |  |  |
| **organic polymers**  **ORGANIC POLYMERS** | **Chitosan** | Delivery vehicles with  mucoadhesive properties, TLR4 and other PRRs | H1N1, H3N2 or H5N1 split viruses | serum/mucosal Abs  systemic type 1/type 2 cytokine-producing cells; local cellular immunity | ND | mouse | (52-54) | phase I  trivalent split vaccine:  safe, well tolerated, immunogenic (serum humoral responses)  no evaluation of adjuvant effect  (55) | safe in humans and efficient in pre-clinical tests (enhancement of humoral and cellular immunity) |
|  |  |  | H5N1 split virus | serum Abs | Homologous  (H5N1) | ferret | (56) |  |  |
|  |  |  | H9N2 M2 | serum/mucosal Abs | Homologous (H9N2) | mouse | (57) |  |  |
|  | **γ-PGA Polymers**  **γ-PGA Polymers** | Delivery vehicles with  mucoadhesive properties | H1N1 split virus | serum/mucosal Abs  systemic type 1/type 2 cytokine-producing cells | Homologous  (H1N1) Heterovariant (H1N1) | mouse | (58) | N/A | efficient in pre-clinical tests (enhancement of humoral and cellular immunity) |
|  |  |  | fusion protein M2-HA2  plus MPLA and QS21  M2: H1N1/H5N1/H9N2 consensus sequence  HA2: portion of H5N1 HA2 | serum/mucosal Abs  systemic type 1 cytokine-producing cells | Homologous, Heterologous  (H1N1, H5N1, H5N2, H7N3, H9N2) | mouse | (59) |  |  |
|  |  |  | fusion protein  M2-HA2-CTA1  chitosan-coated γ-PGA nanoparticles  M2: H1N1/H5N1/H9N2 consensus sequence  HA2: portion of H5N1 HA2 | serum/mucosal Abs  systemic type 1/type 2 cytokine-producing cells | Homologous, Heterologous  (H1N1, H5N1, H5N2, H7N3, H9N2) | mouse | (4) |  |  |
|  | **PLGA** | Delivery vehicles | H9N2 WIV  ± PEI-CpG-ODN | serum/mucosal Abs | ND | chicken | (60) | N/A | efficient in pre-clinical tests (enhancement of humoral immunity), approved by regulatory agencies for delivery of pharmaceuticals in humans |
|  |  |  | H4N6 WIV  chitosan-coated PLGA nanoparticles encapsulating PEI-CpG-ODN | serum/mucosal Abs | ND | chicken | (61) |  |  |
|  | **PEI** | Delivery vehicles with  mucoadhesive properties | H5N1 HA pDNA | serum/mucosal Abs  local/systemic type 1 cytokine-producing cells | Homologous (H5N1) | mouse | (62) | N/A | efficient in pre-clinical tests (enhancement of humoral immunity), possibility to generate less toxic PEI derivatives |
|  |  |  | H9N2 WIV | serum/mucosal Abs | ND | mouse | (63) |  |  |
|  |  |  | H7N9 HA1 | serum/mucosal Abs | Homologous (H7N9)  [mucosal viral loads] | chicken | (16) |  |  |
| **inorganic nano**  **particles** | **Gold Nano-**  **particles** | Delivery vehicles | H3N2 trimeric HA/flagellin | serum/mucosal Abs  local type 1 cytokine-producing cells  systemic type 1/type 2/type 17 cytokine-producing cells  systemic CTLs | Homologous (H3N2) | mouse | (64) | N/A | efficient in pre-clinical tests (enhancement of humoral and cellular immunity),  immunologically inert, tight control over size and shape  /  potential toxicity depending on stabilizers/surface modifiers |
|  |  |  | M2e “human”  consensus sequence ± CpG-ODN | serum Abs | Homologous  (H1N1) | mouse | (65) |  |  |
| **cytokines** | **Type I IFNs** | IFN-1R | H1N1 split virus | serum/mucosal Abs | Homologous (H1N1) | mouse | (66) | phase I  H1N1 IIV with type I IFNs:  no adjuvant effect on serum and mucosal humoral responses  (67) | efficient in pre-clinical tests (enhancement of humoral and cellular immunity), innate endogenous molecules likely to be less toxic than pathogen-derived or synthetic adjuvants  /  short half-life, pleiotropic effects likely to induce  undesired effects |
|  | **IL-1 family cytokines** | IL-1 receptor family | H1N1 HA | serum/mucosal Abs  systemic type 1/type 2 cytokine-producing cells  systemic CTLs | Heterovariant  (H1N1) | mouse | (68) |  |  |
|  | **CCL28** | CCR3/CCR10 | H3N2 (HA/M1) VLPs | serum/mucosal Abs | Homologous (H3N2)  Heterovariant (H3N2) | mouse | (69) |  |  |
|  | **IL-12** | IL-12R | H1N1 HA/NA | serum/mucosal Abs | Homologous (H1N1) | mouse | (70) |  |  |

^a^ Significant enhancement of the immune responses of animals of the adjuvanted-vaccine group in comparison with animals of the unadjuvanted-vaccine group.

^b^ Significant enhancement of the survival of animals of the adjuvanted-vaccine group in comparison with animals of the unadjuvanted-vaccine group against intranasal IAV challenge.

Abbreviations:

Abs: antibodies; Ags: antigens; CCL: (C-C motif) ligand; CCR: (C-C motif) receptor; c-di-AMP: cyclic-di-adenosine monophosphate; c-di-GMP: cyclic-di-guanosine monophosphate; cGAMP: 2’,3’-cyclic-guanosine monophosphate-adenosine monophosphate; CpG-ODN: synthetic oligodeoxynucleotides composed of unmethylated CpG motifs; CT: cholera toxin; CTA1: A1 subunit of the cholera toxin; CTA1-DD: CTA1 fused to a synthetic dimer of the Ig binding D domain (DD) from *Staphylococcus aureus* protein A; CTLs: cytotoxic T lymphocytes; HA: hemagglutinin; HA1: globular head domain of hemagglutinin; HA2: stalk domain of hemagglutinin; IAV: influenza A virus; IBV: influenza B virus; IFNs: interferons; Ig: immunoglobulins; IL: interleukin; ISCOMs: immune stimulating complexes; LAIV: live-attenuated influenza viruses; LT: *Escherichia coli* heat-labile toxin; M1: matrix protein 1; M2(e): (ectodomain of the) matrix protein 2; MPLA: monophosphoryl lipid A; NA: neuraminidase; N/A: not available; ND: not determined; NP: nucleoprotein; PEI: polyethyleneimine; γ-PGA: poly (γ-glutamic acid); PLGA: poly (D, L-lactide-co-glycolide); poly (I:C): polyinosine-polycytidylic acid; PRR: pattern recognition receptor; STING: stimulator of interferon genes; TLR: Toll-like receptor; VLPs: virus-like particles; WIV: whole inactivated viruses.

References:

1. Quan FS, Compans RW, Nguyen HH, Kang SM. Induction of heterosubtypic immunity to influenza virus by intranasal immunization. *J Virol* (2008) 82(3):1350-9. doi: 10.1128/JVI.01615-07

2. Lycke N, Lebrero-Fernandez C. ADP-ribosylating enterotoxins as vaccine adjuvants. *Curr Opin Pharmacol* (2018) 41:42-51. doi: 10.1016/j.coph.2018.03.015

3. Tumpey TM, Renshaw M, Clements JD, Katz JM. Mucosal delivery of inactivated influenza vaccine induces B-cell-dependent heterosubtypic cross-protection against lethal influenza A H5N1 virus infection. *J Virol* (2001) 75(11):5141-50. doi: 10.1128/JVI.75.11.5141-5150.2001

4. Chowdhury MYE, Kim TH, Uddin MB, Kim JH, Hewawaduge CY, Ferdowshi Z, et al. Mucosal vaccination of conserved sM2, HA2 and cholera toxin subunit A1 (CTA1) fusion protein with poly γ-glutamate/chitosan nanoparticles (PC NPs) induces protection against divergent influenza subtypes. *Vet Microbiol* (2017) 201:240-51. doi: 10.1016/j.vetmic.2017.01.020

5. De Filette M, Ramne A, Birkett A, Lycke N, Lowenadler B, Min Jou W, et al. The universal influenza vaccine M2e-HBc administered intranasally in combination with the adjuvant CTA1-DD provides complete protection. *Vaccine* (2006) 24(5):544-51. doi: 10.1016/j.vaccine.2005.08.061

6. Helgeby A, Robson NC, Donachie AM, Beackock-Sharp H, Lovgren K, Schon K, et al. The combined CTA1-DD/ISCOM adjuvant vector promotes priming of mucosal and systemic immunity to incorporated antigens by specific targeting of B cells. *Journal of immunology* (2006) 176(6):3697-706

7. Liang J, Fu J, Kang H, Lin J, Yu Q, Yang Q. Comparison of 3 kinds of Toll-like receptor ligands for inactivated avian H5N1 influenza virus intranasal immunization in chicken. *Poult Sci* (2013) 92(10):2651-60. doi: 10.3382/ps.2013-03193

8. Overton ET, Goepfert PA, Cunningham P, Carter WA, Horvath J, Young D, et al. Intranasal seasonal influenza vaccine and a TLR-3 agonist, rintatolimod, induced cross-reactive IgA antibody formation against avian H5N1 and H7N9 influenza HA in humans. *Vaccine* (2014) 32(42):5490-5. doi: 10.1016/j.vaccine.2014.07.078

9. Takaki H, Kure S, Oshiumi H, Sakoda Y, Suzuki T, Ainai A, et al. Toll-like receptor 3 in nasal CD103^+^ dendritic cells is involved in immunoglobulin A production. *Mucosal Immunol* (2018) 11(1):82-96. doi: 10.1038/mi.2017.48

10. Goff PH, Eggink D, Seibert CW, Hai R, Martinez-Gil L, Krammer F, et al. Adjuvants and immunization strategies to induce influenza virus hemagglutinin stalk antibodies. *PLoS One* (2013) 8(11):e79194. doi: 10.1371/journal.pone.0079194

11. Ichinohe T, Tamura S, Kawaguchi A, Ninomiya A, Imai M, Itamura S, et al. Cross-protection against H5N1 influenza virus infection is afforded by intranasal inoculation with seasonal trivalent inactivated influenza vaccine. *J Infect Dis* (2007) 196(9):1313-20. doi: 10.1086/521304

12. Skountzou I, Martin Mdel P, Wang B, Ye L, Koutsonanos D, Weldon W, et al. Salmonella flagellins are potent adjuvants for intranasally administered whole inactivated influenza vaccine. *Vaccine* (2010) 28(24):4103-12. doi: 10.1016/j.vaccine.2009.07.058

13. Hong SH, Byun YH, Nguyen CT, Kim SY, Seong BL, Park S, et al. Intranasal administration of a flagellin-adjuvanted inactivated influenza vaccine enhances mucosal immune responses to protect mice against lethal infection. *Vaccine* (2012) 30(2):466-74. doi: 10.1016/j.vaccine.2011.10.058

14. Wang BZ, Gill HS, He C, Ou C, Wang L, Wang YC, et al. Microneedle delivery of an M2e-TLR5 ligand fusion protein to skin confers broadly cross-protective influenza immunity. *J Control Release* (2014) 178:1-7. doi: 10.1016/j.jconrel.2014.01.002

15. Wang BZ, Xu R, Quan FS, Kang SM, Wang L, Compans RW. Intranasal immunization with influenza VLPs incorporating membrane-anchored flagellin induces strong heterosubtypic protection. *PLoS One* (2010) 5(11):e13972. doi: 10.1371/journal.pone.0013972

16. Song L, Xiong D, Song H, Wu L, Zhang M, Kang X, et al. Mucosal and systemic immune responses to influenza H7N9 antigen HA1-2 co-delivered intranasally with flagellin or polyethyleneimine in mice and chickens. *Front Immunol* (2017) 8:326. doi: 10.3389/fimmu.2017.00326

17. Qin T, Yin Y, Yu Q, Huang L, Wang X, Lin J, et al. CpG oligodeoxynucleotides facilitate delivery of whole inactivated H9N2 influenza virus via transepithelial dendrites of dendritic cells in nasal mucosa. *J Virol* (2015) 89(11):5904-18. doi: 10.1128/JVI.00296-15

18. Fu J, Liang J, Kang H, Lin J, Yu Q, Yang Q. Effects of different CpG oligodeoxynucleotides with inactivated avian H5N1 influenza virus on mucosal immunity of chickens. *Poult Sci* (2013) 92(11):2866-75. doi: 10.3382/ps.2013-03205. PubMed PMID: 24135589

19. Ebensen T, Debarry J, Pedersen GK, Blazejewska P, Weissmann S, Schulze K, et al. Mucosal administration of cycle-di-nucleotide-adjuvanted virosomes efficiently induces protection against influenza H5N1 in mice. *Front Immunol* (2017) 8:1223. doi: 10.3389/fimmu.2017.01223

20. Sanchez MV, Ebensen T, Schulze K, Cargnelutti D, Blazejewska P, Scodeller EA, et al. Intranasal delivery of influenza rNP adjuvanted with c-di-AMP induces strong humoral and cellular immune responses and provides protection against virus challenge. *PLoS One* (2014) 9(8):e104824. doi: 10.1371/journal.pone.0104824

21. Takaki H, Takashima K, Oshiumi H, Ainai A, Suzuki T, Hasegawa H, et al. cGAMP promotes germinal center formation and production of IgA in nasal-associated lymphoid tissue. *Med Sci (Basel)* (2017) 5(4):35. doi: 10.3390/medsci5040035

22. Madhun AS, Haaheim LR, Nostbakken JK, Ebensen T, Chichester J, Yusibov V, et al. Intranasal c-di-GMP-adjuvanted plant-derived H5 influenza vaccine induces multifunctional Th1 CD4^+^ cells and strong mucosal and systemic antibody responses in mice. *Vaccine* (2011) 29(31):4973-82. doi: 10.1016/j.vaccine.2011.04.094

23. Plante M, Jones T, Allard F, Torossian K, Gauthier J, St-Felix N, et al. Nasal immunization with subunit proteosome influenza vaccines induces serum HAI, mucosal IgA and protection against influenza challenge. *Vaccine* (2001) 20(1-2):218-25

24. Lambkin-Williams R, Gelder C, Broughton R, Mallett CP, Gilbert AS, Mann A, et al. An intranasal proteosome-adjuvanted trivalent influenza vaccine is safe, immunogenic & efficacious in the human viral influenza challenge model. Serum IgG & mucosal IgA are important correlates of protection against illness associated with infection. *PLoS One* (2016) 11(12):e0163089. doi: 10.1371/journal.pone.0163089

25. Jones T, Allard F, Cyr SL, Tran SP, Plante M, Gauthier J, et al. A nasal Proteosome influenza vaccine containing baculovirus-derived hemagglutinin induces protective mucosal and systemic immunity. *Vaccine* (2003) 21(25-26):3706-12

26. Jones T, Cyr S, Allard F, Bellerose N, Lowell GH, Burt DS. Protollin: a novel adjuvant for intranasal vaccines. *Vaccine* (2004) 22(27-28):3691-7. doi: 10.1016/j.vaccine.2004.03.035

27. Saluja V, Amorij JP, van Roosmalen ML, Leenhouts K, Huckriede A, Hinrichs WL, et al. Intranasal delivery of influenza subunit vaccine formulated with GEM particles as an adjuvant. *AAPS J* (2010) 12(2):109-16. doi: 10.1208/s12248-009-9168-2

28. de Haan A, Haijema BJ, Voorn P, Meijerhof T, van Roosmalen ML, Leenhouts K. Bacterium-like particles supplemented with inactivated influenza antigen induce cross-protective influenza-specific antibody responses through intranasal administration. *Vaccine* (2012) 30(32):4884-91. doi: 10.1016/j.vaccine.2012.04.032

29. Van Braeckel-Budimir N, Haijema BJ, Leenhouts K. Bacterium-like particles for efficient immune stimulation of existing vaccines and new subunit vaccines in mucosal applications. *Front Immunol* (2013) 4:282. doi: 10.3389/fimmu.2013.00282

30. Lee TY, Kim CU, Bae EH, Seo SH, Jeong DG, Yoon SW, et al. Outer membrane vesicles harboring modified lipid A moiety augment the efficacy of an influenza vaccine exhibiting reduced endotoxicity in a mouse model. *Vaccine* (2017) 35(4):586-95. doi: 10.1016/j.vaccine.2016.12.025

31. Joseph A, Louria-Hayon I, Plis-Finarov A, Zeira E, Zakay-Rones Z, Raz E, et al. Liposomal immunostimulatory DNA sequence (ISS-ODN): an efficient parenteral and mucosal adjuvant for influenza and hepatitis B vaccines. *Vaccine* (2002) 20(27-28):3342-54

32. Even-Or O, Samira S, Ellis R, Kedar E, Barenholz Y. Adjuvanted influenza vaccines. *Expert Rev Vaccines* (2013) 12(9):1095-108. doi: 10.1586/14760584.2013.825445

33. Bernasconi V, Norling K, Bally M, Hook F, Lycke NY. Mucosal vaccine development based on liposome technology. *J Immunol Res* (2016) 2016:5482087. doi: 10.1155/2016/5482087.

34. Moser C, Muller M, Kaeser MD, Weydemann U, Amacker M. Influenza virosomes as vaccine adjuvant and carrier system. *Expert Rev Vaccines* (2013) 12(7):779-91. doi: 10.1586/14760584.2013.811195

35. Corthesy B, Bioley G. Lipid-based particles: Versatile delivery systems for mucosal vaccination against infection. *Front Immunol* (2018) 9:431. doi: 10.3389/fimmu.2018.00431

36. Christensen D, Foged C, Rosenkrands I, Lundberg CV, Andersen P, Agger EM, et al. CAF01 liposomes as a mucosal vaccine adjuvant: *in vitro* and *in vivo* investigations. *Int J Pharm* (2010) 390(1):19-24. doi: 10.1016/j.ijpharm.2009.10.043

37. Qu W, Li N, Yu R, Zuo W, Fu T, Fei W, et al. Cationic DDA/TDB liposome as a mucosal vaccine adjuvant for uptake by dendritic cells *in vitro* induces potent humoural immunity. *Artif Cells Nanomed Biotechnol* (2018) 46(sup1):852-60. doi: 10.1080/21691401.2018.1438450

38. Joseph A, Itskovitz-Cooper N, Samira S, Flasterstein O, Eliyahu H, Simberg D, et al. A new intranasal influenza vaccine based on a novel polycationic lipid--ceramide carbamoyl-spermine (CCS) I. Immunogenicity and efficacy studies in mice. *Vaccine* (2006) 24(18):3990-4006. doi: 10.1016/j.vaccine.2005.12.017

39. Wang D, Christopher ME, Nagata LP, Zabielski MA, Li H, Wong JP, et al. Intranasal immunization with liposome-encapsulated plasmid DNA encoding influenza virus hemagglutinin elicits mucosal, cellular and humoral immune responses. *J Clin Virol* (2004) 31 Suppl 1:S99-106. doi: 10.1016/j.jcv.2004.09.013

40. Eliasson DG, Helgeby A, Schon K, Nygren C, El-Bakkouri K, Fiers W, et al. A novel non-toxic combined CTA1-DD and ISCOMS adjuvant vector for effective mucosal immunization against influenza virus. *Vaccine* (2011) 29(23):3951-61. doi: 10.1016/j.vaccine.2011.03.090

41. Drane D, Gittleson C, Boyle J, Maraskovsky E. ISCOMATRIX adjuvant for prophylactic and therapeutic vaccines. *Expert Rev Vaccines* (2007) 6(5):761-72. doi: 10.1586/14760584.6.5.761

42. Kimoto T, Mizuno D, Takei T, Kunimi T, Ono S, Sakai S, et al. Intranasal influenza vaccination using a new synthetic mucosal adjuvant SF-10: induction of potent local and systemic immunity with balanced Th1 and Th2 responses. *Influenza Other Respir Viruses* (2013) 7(6):1218-26. doi: 10.1111/irv.12124

43. Kim H, Kimoto T, Sakai S, Takahashi E, Kido H. Adjuvanting influenza hemagglutinin vaccine with a human pulmonary surfactant-mimicking synthetic compound SF-10 induces local and systemic cell-mediated immunity in mice. *PLoS One* (2018) 13(1):e0191133. doi: 10.1371/journal.pone.0191133

44. Mizuno D, Kimoto T, Sakai S, Takahashi E, Kim H, Kido H. Induction of systemic and mucosal immunity and maintenance of its memory against influenza A virus by nasal vaccination using a new mucosal adjuvant SF-10 derived from pulmonary surfactant in young cynomolgus monkeys. *Vaccine* (2016) 34(16):1881-8. doi: 10.1016/j.vaccine.2016.02.06

45. Falkeborn T, Brave A, Larsson M, Akerlind B, Schroder U, Hinkula J. Endocine, N3OA and N3OASq; three mucosal adjuvants that enhance the immune response to nasal influenza vaccination. *PLoS One* (2013) 8(8):e70527. doi: 10.1371/journal.pone.0070527

46. Das SC, Hatta M, Wilker PR, Myc A, Hamouda T, Neumann G, et al. Nanoemulsion W_80_5EC improves immune responses upon intranasal delivery of an inactivated pandemic H1N1 influenza vaccine. *Vaccine* (2012) 30(48):6871-7. doi: 10.1016/j.vaccine.2012.09.007

47. Stanberry LR, Simon JK, Johnson C, Robinson PL, Morry J, Flack MR, et al. Safety and immunogenicity of a novel nanoemulsion mucosal adjuvant W_80_5EC combined with approved seasonal influenza antigens. *Vaccine* (2012) 30(2):307-16. doi: 10.1016/j.vaccine.2011.10.094

48. Hamouda T, Sutcliffe JA, Ciotti S, Baker JR, Jr. Intranasal immunization of ferrets with commercial trivalent influenza vaccines formulated in a nanoemulsion-based adjuvant. *Clin Vaccine Immunol* (2011) 18(7):1167-75. doi: 10.1128/CVI.00035-11

49. Xia M, Tan M, Wei C, Zhong W, Wang L, McNeal M, et al. A candidate dual vaccine against influenza and noroviruses. *Vaccine* (2011) 29(44):7670-7. doi: 10.1016/j.vaccine.2011.07.139

50. Wang L, Wang YC, Feng H, Ahmed T, Compans RW, Wang BZ. Virus-like particles containing the tetrameric ectodomain of influenza matrix protein 2 and flagellin induce heterosubtypic protection in mice. *Biomed Res Int* (2013) 2013:686549. doi: 10.1155/2013/686549

51. Herve PL, Raliou M, Bourdieu C, Dubuquoy C, Petit-Camurdan A, Bertho N, et al. A novel subnucleocapsid nanoplatform for mucosal vaccination against influenza virus that targets the ectodomain of matrix protein 2. *J Virol* (2014) 88(1):325-38. doi: 10.1128/JVI.01141-13

52. Liu Q, Zheng X, Zhang C, Shao X, Zhang X, Zhang Q, et al. Conjugating influenza A (H1N1) antigen to N-trimethylaminoethylmethacrylate chitosan nanoparticles improves the immunogenicity of the antigen after nasal administration. *J Med Virol* (2015) 87(11):1807-15. doi: 10.1002/jmv.24253

53. Amidi M, Romeijn SG, Verhoef JC, Junginger HE, Bungener L, Huckriede A, et al. N-trimethyl chitosan (TMC) nanoparticles loaded with influenza subunit antigen for intranasal vaccination: biological properties and immunogenicity in a mouse model. *Vaccine* (2007) 25(1):144-53. doi: 10.1016/j.vaccine.2006.06.086

54. Wu Y, Wei W, Zhou M, Wang Y, Wu J, Ma G, et al. Thermal-sensitive hydrogel as adjuvant-free vaccine delivery system for H5N1 intranasal immunization. *Biomaterials* (2012) 33(7):2351-60. doi: 10.1016/j.biomaterials.2011.11.068

55. Read RC, Naylor SC, Potter CW, Bond J, Jabbal-Gill I, Fisher A, et al. Effective nasal influenza vaccine delivery using chitosan. *Vaccine* (2005) 23(35):4367-74. doi: 10.1016/j.vaccine.2005.04.021

56. Mann AJ, Noulin N, Catchpole A, Stittelaar KJ, de Waal L, Veldhuis Kroeze EJ, et al. Intranasal H5N1 vaccines, adjuvanted with chitosan derivatives, protect ferrets against highly pathogenic influenza intranasal and intratracheal challenge. *PLoS One* (2014) 9(5):e93761. doi: 10.1371/journal.pone.0093761

57. Sui Z, Chen Q, Wu R, Zhang H, Zheng M, Wang H, et al. Cross-protection against influenza virus infection by intranasal administration of M2-based vaccine with chitosan as an adjuvant. *Arch Virol* (2010) 155(4):535-44. doi: 10.1007/s00705-010-0621-4

58. Okamoto S, Matsuura M, Akagi T, Akashi M, Tanimoto T, Ishikawa T, et al. Poly(γ-glutamic acid) nano-particles combined with mucosal influenza virus hemagglutinin vaccine protects against influenza virus infection in mice. *Vaccine* (2009) 27(42):5896-905. doi: 10.1016/j.vaccine.2009.07.037

59. Noh HJ, Chowdhury MY, Cho S, Kim JH, Park HS, Kim CJ, et al. Programming of influenza vaccine broadness and persistence by mucoadhesive polymer-based adjuvant systems. *Journal of immunology* (2015) 195(5):2472-82. doi: 10.4049/jimmunol.1500492

60. Singh SM, Alkie TN, Abdelaziz KT, Hodgins DC, Novy A, Nagy E, et al. Characterization of immune responses to an inactivated avian influenza virus vaccine adjuvanted with nanoparticles containing CpG ODN. *Viral Immunol* (2016) 29(5):269-75. doi: 10.1089/vim.2015.0144

61. Alkie TN, Yitbarek A, Taha-Abdelaziz K, Astill J, Sharif S. Characterization of immunogenicity of avian influenza antigens encapsulated in PLGA nanoparticles following mucosal and subcutaneous delivery in chickens. *PLoS One* (2018) 13(11):e0206324. doi: 10.1371/journal.pone.0206324

62. Torrieri-Dramard L, Lambrecht B, Ferreira HL, Van den Berg T, Klatzmann D, Bellier B. Intranasal DNA vaccination induces potent mucosal and systemic immune responses and cross-protective immunity against influenza viruses. *Mol Ther* (2011) 19(3):602-11. doi: 10.1038/mt.2010.222

63. Qin T, Yin Y, Huang L, Yu Q, Yang Q. H9N2 influenza whole inactivated virus combined with polyethyleneimine strongly enhances mucosal and systemic immunity after intranasal immunization in mice. *Clin Vaccine Immunol* (2015) 22(4):421-9. doi: 10.1128/CVI.00778-14

64. Wang C, Zhu W, Luo Y, Wang BZ. Gold nanoparticles conjugating recombinant influenza hemagglutinin trimers and flagellin enhanced mucosal cellular immunity. *Nanomedicine* (2018) 14(4):1349-60. doi: 10.1016/j.nano.2018.03.007

65. Tao W, Ziemer KS, Gill HS. Gold nanoparticle-M2e conjugate coformulated with CpG induces protective immunity against influenza A virus. *Nanomedicine (Lond)* (2014) 9(2):237-51. doi: 10.2217/nnm.13.58

66. Bracci L, Canini I, Puzelli S, Sestili P, Venditti M, Spada M, et al. Type I IFN is a powerful mucosal adjuvant for a selective intranasal vaccination against influenza virus in mice and affects antigen capture at mucosal level. *Vaccine* (2005) 23(23):2994-3004. doi: 10.1016/j.vaccine.2004.12.006

67. Couch RB, Atmar RL, Cate TR, Quarles JM, Keitel WA, Arden NH, et al. Contrasting effects of type I interferon as a mucosal adjuvant for influenza vaccine in mice and humans. *Vaccine* (2009) 27(39):5344-8. doi: 10.1016/j.vaccine.2009.06.084

68. Kayamuro H, Yoshioka Y, Abe Y, Arita S, Katayama K, Nomura T, et al. Interleukin-1 family cytokines as mucosal vaccine adjuvants for induction of protective immunity against influenza virus. *J Virol* (2010) 84(24):12703-12. doi: 10.1128/JVI.01182-10

69. Mohan T, Kim J, Berman Z, Wang S, Compans RW, Wang BZ. Co-delivery of GPI-anchored CCL28 and influenza HA in chimeric virus-like particles induces cross-protective immunity against H3N2 viruses. *J Control Release* (2016) 233:208-19. doi: 10.1016/j.jconrel.2016.05.021

70. Arulanandam BP, O'Toole M, Metzger DW. Intranasal interleukin-12 is a powerful adjuvant for protective mucosal immunity. *J Infect Dis* (1999) 180(4):940-9. doi: 10.1086/314996
